# MiR-CLIP reveals *iso*-miR selective regulation in the miR-124 targetome

**DOI:** 10.1093/nar/gkaa1117

**Published:** 2020-12-09

**Authors:** Yuluan Wang, Charlotte Soneson, Anna L Malinowska, Artur Laski, Souvik Ghosh, Alexander Kanitz, Luca F R Gebert, Mark D Robinson, Jonathan Hall

**Affiliations:** Institute of Pharmaceutical Sciences, ETH Zurich, Vladimir-Prelog-Weg 4, 8093 Zurich, Switzerland; Department of Molecular Life Sciences and SIB Swiss Institute of Bioinformatics, University of Zurich, 8057, Zurich, Switzerland; Institute of Pharmaceutical Sciences, ETH Zurich, Vladimir-Prelog-Weg 4, 8093 Zurich, Switzerland; Institute of Pharmaceutical Sciences, ETH Zurich, Vladimir-Prelog-Weg 4, 8093 Zurich, Switzerland; Biozentrum, University of Basel, 4056 Basel, Switzerland; Biozentrum, University of Basel, 4056 Basel, Switzerland; Department of Integrative Structural and Computational Biology, The Scripps Research Institute, La Jolla, CA 92037, USA; Department of Molecular Life Sciences and SIB Swiss Institute of Bioinformatics, University of Zurich, 8057, Zurich, Switzerland; Institute of Pharmaceutical Sciences, ETH Zurich, Vladimir-Prelog-Weg 4, 8093 Zurich, Switzerland

## Abstract

Many microRNAs regulate gene expression via atypical mechanisms, which are difficult to discern using native cross-linking methods. To ascertain the scope of non-canonical miRNA targeting, methods are needed that identify all targets of a given miRNA. We designed a new class of miR-CLIP probe, whereby psoralen is conjugated to the 3p arm of a pre-microRNA to capture targetomes of miR-124 and miR-132 in HEK293T cells. Processing of pre-miR-124 yields miR-124 and a 5′-extended isoform, *iso*-miR-124. Using miR-CLIP, we identified overlapping targetomes from both isoforms. From a set of 16 targets, 13 were differently inhibited at mRNA/protein levels by the isoforms. Moreover, delivery of pre-miR-124 into cells repressed these targets more strongly than individual treatments with miR-124 and *iso*-miR-124, suggesting that isomirs from one pre-miRNA may function synergistically. By mining the miR-CLIP targetome, we identified nine G-bulged target-sites that are regulated at the protein level by miR-124 but not isomiR-124. Using structural data, we propose a model involving AGO2 helix-7 that suggests why only miR-124 can engage these sites. In summary, access to the miR-124 targetome via miR-CLIP revealed for the first time how heterogeneous processing of miRNAs combined with non-canonical targeting mechanisms expand the regulatory range of a miRNA.

## INTRODUCTION

MiRNAs are short RNAs that regulate post-transcriptional gene expression. Over the last decade the understanding of miRNA biology has been advanced by comprehensive sequencing and annotation programs (1), through mapping of their expression profiles (2), by clarifying their biogenesis and metabolism (3,4), as well as by studies with model organisms (2) that assigned physiological functions and the roles of miRNAs in disease (5). However, without the identification of a miRNA’s targetome, knowledge of its function remains incomplete.

MiRNA biogenesis is a complex process that begins with transcription of the primary miRNA performed by RNA polymerase II. This action yields a hairpin structure comprising two mature miRNA strands embedded in the 5p and 3p arms of the stem. The transcript is cleaved to the precursor miRNA (pre-miRNA) by a complex of drosha ribonuclease III (DROSHA) and DiGeorge syndrome chromosomal region 8 (DGCR8). Pre-miRNAs are then transported to the cytoplasm where, DICER (Dicer) and the transactivation response element RNA-binding protein (TRBP) excise the loop by cleaving at the 3′ end of the 5p strand, and at the 5′ end of the 3p strand. Cleavage of miRNA precursors by Drosha and Dicer is heterogeneous and yields a population of miRNA isoforms (isomiRs) that vary at their termini by one or more nucleotides (4,6,7). The double-stranded miRNA is then bound by an Argonaute protein (AGO)—core of the miRNA-induced silencing complex (miRISC)—which anchors the guide strand and primes it for targeting.

MiRNA sequences are grouped into families, each of which may suppress expression of hundreds of mRNA targets (8). The canonical mechanism of miRNA action involves base-pairing of its seed region to conserved complementary sites in mRNA 3′UTRs (9,10). The analysis of large data sets yielded principles that link the degree of target suppression to their distinct seed-binding regions (11,12). Variations at the 5′ terminus of a miRNA, which are due to the aforementioned heterogeneous processing, produce shifted seed ‘registers’ and potentially different targeting profiles. This intriguing aspect of miRNA biogenesis has far reaching consequences but is rarely investigated, partly because it is challenging in the cellular context to differentiate between the properties of two RNAs that differ by a single nucleotide.

Computational methods and native cross-linking and immunoprecipitation (CLIP) techniques have been instrumental for the identification of miRNA targetomes. However, these genome-wide approaches are less useful where seed-target complementarity is only partially, or not at all, implicated in a regulation (2,11,13–22). Furthermore, data derived from CLIP experiments performed under native conditions, suggests that most miRNA–mRNA interactions are non-canonical (18,23), and may depend upon miRNA-target binding in the central region (13,14) or at the 3′ end of the miRNA (8,22). Some have argued that much of the CLIP data simply represents snapshots of transient, non-functional RNA–RNA contacts in the cell (12,24). Unfortunately, factors such as low signal-to-noise ratio, low read-depth of miRNA–mRNA events and uncertainty about the miRNA family member or isomiR implicated in cross-linking events, generally complicate follow-up studies from these native methods.

Recently, we described a new technique that is able to identify the targetome of a miRNA (21). MiR-CLIP (miRNA cross-linking and immunoprecipitation) uses state-of-the-art RNA synthesis for preparation of a pre-miRNA probe that is site-specifically equipped in its 5p strand with psoralen and biotin groups. In cells, the probe is processed into a mature miRNA, which then cross-links to its targets in RISC upon mild irradiation. Finally, streptavidin-aided enrichment of RNA obtained after AGO2 pulldown enables isolation of the specific targets of the miRNA. The key advantage of miR-CLIP over conventional CLIP methods is that it captures both canonical and non-canonical targets of a sequence-defined miRNA. This provides high confidence in the hits and facilitates analysis and design of validation experiments. Using miR-CLIP, we discovered that miR-106a-5p regulates, and is regulated by, the long non-coding RNA H19 (21).

In this study, we extended miR-CLIP to the 3p miRNAs, miR-132 and miR-124, with the introduction of psoralen and biotin in the 3p arm of a miRNA precursor. Deep sequencing of cDNAs generated from miR-CLIP RNA libraries from human embryonic kidney cells (HEK293T), identified reproducibly dozens of miR-132 and -124 targets, which were validated at the mRNA level. This included a set of 16 mRNAs that were highly destabilized by miR-124 and that are depleted in brain. We validated these on a functional level by qPCR and shotgun proteomics by LC-MS analysis (25) after miRNA transfection into cells. We confirmed that pre-miR-124 produces two 5′ isomiRs (26), which are able to regulate distinct but overlapping targetomes derived from seed registers that are shifted by a single nucleotide. Furthermore, upon mining the miR-CLIP targetome we identified and then validated the regulation of nine mRNAs at sites containing G-bulges. Surprisingly, these were suppressed specifically by miR-124, but not by *iso*-miR-124. Using structural data, we propose a model involving helix-7 of AGO2, which explains why miR-124 alone can engage the target. Taken together, this shows how miRNA and Argonautes work together to create a non-canonical interaction that enables isomiR-specific targeting.

## MATERIALS AND METHODS

### Post-synthetic modification of 2′-O-propargyl–substituted oligoribonucleotides by CuAAC

Copper(I)-catalyzed azide-alkyne cycloaddition (CuAAC) reaction between alkynyl-modified oligoribonucleotides and azide-bearing biotin or psoralen (trioxsalen) was performed as described previously (21,27). After solid phase synthesis, the CPG containing the alkynyl-modified RNA was suspended in 300 μl of H_2_O/PBS (1:1) mixture. Subsequently, the appropriate azide (20 eq, 1 μmol in 60 μl of DMF), TBTA (10 eq, 500 nmol, 0.27 mg in 20 μl of DMF), Na-ascorbate (10 eq, 500 nmol, 10 μl of a solution containing 10 mg in 1 ml of H_2_O) and CuSO_4_*5H_2_O (1 eq, 50 nmol, 10 μl of a solution containing 12.5 mg in 10 ml of H_2_O) were added to the suspension in this order. All solutions were freshly prepared prior to use. The reaction mixture was shaken (1400 rpm) overnight at 45°C in an Eppendorf shaker (under Argon in case of reaction with biotin azide). The CPG was filtered off and washed three times with 0.5 ml of each: DMF, 0.1 N aqueous EDTA, DMF, ACN, CHCl_3_ and dried under vacuum. Afterward, post-synthetically modified oligoribonucleotides were deprotected according to the standard procedure (described above).

### Cell culture

HEK293T cells (ATCC® CRL-3216™, Wesel, DE) were cultivated in Dulbecco’s Modified Eagle’s medium (Gibco, Invitrogen, Basel, CH) supplemented with 10% FBS (Gibco, Invitrogen, Basel, CH).

### Luciferase reporter assays

Inserts for reporter plasmids (Supplementary Table S2) were generated by DNA synthesis, then cloned into psiCHECK2 vector (no. C8021, Promega, Dübendorf). HEK293T cells were seeded into 96-well plates. Cells were transfected with indicated concentrations of RNA, using Lipofectamine 2000 (no. 11668019, ThermoFisher Scientific, Basel, CH) according to the manufacturer’s instructions. All transfections were performed in technical triplicates. One day after RNA transfection, 20 ng/well of reporter plasmid were transfected using JetPEI (101-10N, Polyplus, Transfection, Illkirch, FR) according to the manufacturer’s instructions. Two days after the second transfection, cell supernatants were removed and luciferase analysis (Dual-GloR Luciferase Assay System, Promega, Dubendorf, CH)) was performed as per the manufacturer’s instructions with the following changes: Dual-Glo® Luciferase Reagent was diluted 1:1 with H_2_O and added in the volume of 30μL/well, Dual-Glo® Stop & Glo® Reagent was added in the volume of 15 μl/well. Luminescence was measured on a microtiter plate reader (Mithras LB940, Berthold Technologies, Bad Wildbad, DE). Values were normalized against firefly luciferase activity and 0 nM treatment.

### Cell transfection and lysis

About 20% of the fully confluent HEK293T cells from a T75 flask were seeded into 10 cm dishes. Twelve hours after seeding, annealed RNA duplexes consisting of shifted or canonical isomiRs, were transfected at final concentrations of 40 nM, with Lipofectamine RNAiMAX (13778150, Thermo Fisher Scientific) according to the manufacturer’s protocol. Treatment containing transfection reagent without RNA was used as a control. Cells were put on ice, washed with 1× PBS, then scrapped with PBS and pelleted at 200***g***, 4°C for 5 min, After spinning, supernatant was removed and cells were snap-frozen in liquid nitrogen.

### RT-qPCR

HEK293T cells were seeded overnight and RNA was transfected using Lipofectamine 2000 according to manufacturer’s instructions. Cells were lysed at the indicated time point using TRIzol™ Reagent (15596026, Thermo Fisher Scientific), RNA extraction was performed according to the manufacturer’s instructions. RNA was reverse transcribed using the TaqMan™ MicroRNA Reverse Transcription Kit (4366597, Applied Biosystems™). The reverse transcription reaction with the end concentration of: 1× RT buffer, 1× dNTP mix (4 mM), 12.5 μM random hexamers (Microsynth), 12.5 μM oligo(dT)15 (C1101, Promega), 2.5 U multiscribe Reverse transcriptase, 2 U RNAse inhibitor (RNasin, N2115, Promega) was run on C1000 or S1000 Thermal Cycler (Bio-Rad) using the following cycle: 25°C for 10 min, 37°C for 120 min, 85°C for 5 min, 10°C on-hold. Transcript specific primers (Supplementary Table S3) were ordered from Microsynth (Balgach, Switzerland). The SYBR Green PCR was performed in a LightCycler 480 instrument (Roche) with KAPA SYBR® FAST for Roche LightCyler®480 (KK4610, Sigma-Aldrich) following the manufacturer’s instructions. Fold changes are calculated using the 2^−ΔΔCp^ method. Housekeeping genes were used for normalization and mock/negative control treated cells as calibrators.

### *In vitro* photo cross-linking experiments

About 0.2 nmol of trioxsalen-modified RNA and its unmodified counterstrand were mixed, dried, and re-dissolved in 200 μl of annealing buffer (2.5 mM Na_2_HPO_4_, 5 mM NaH_2_PO_4_, 100 mM NaCl and 0.1 mM Na_2_EDTA) so that the final concentration was 1 μM. For annealing, the solution was heated to 95°C, maintained for 5 min, then cooled to room temperature over a period of 2 h. The mixture was put in the open 24-well plate, and was irradiated on ice for 5, 15 or 30 min (365 nm, distance of the solution from the lamp: 5 cm). Then, the sample was directly purified by RP-HPLC (settings as in ‘oligonucleotide synthesis, deprotection and purification’ section) using a gradient 1–60% B in 12 min. Collected fractions were dried, re-dissolved in H_2_O and analyzed by LC-MS (settings as in ‘Oligonucleotide synthesis, deprotection and purification’ section) with a gradient 5–60% B in 14 min.

## RESULTS

### Design of miR-CLIP probes

**Figure 1.**
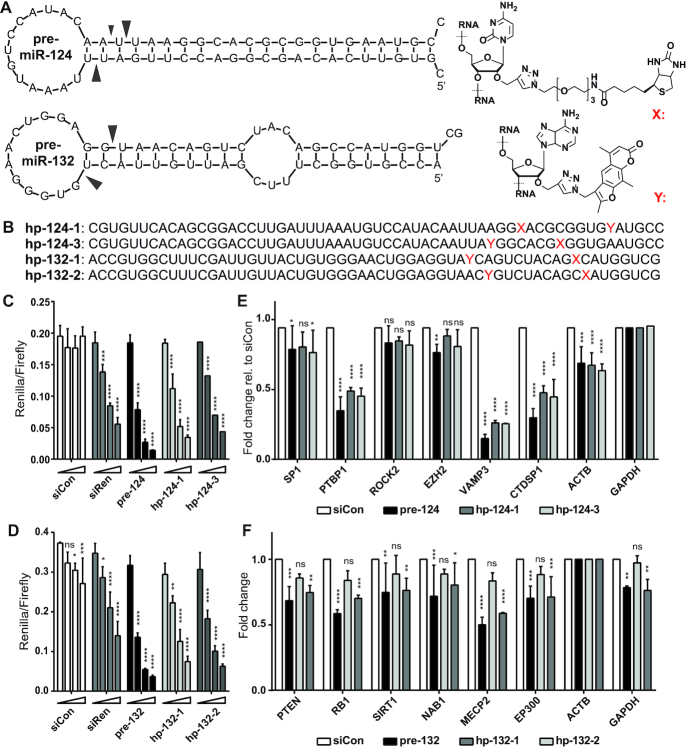
Design and characterization of miR-CLIP probes for miR-124-3p and miR-132-3p. (**A**) Predicted structures (mFold) of pre-miR-124 and pre-miR-132 and structures of the biotin (X) and psoralen (trioxsalen)-(Y) modified bases. Main Dicer-cleavage sites in 5p- and 3p-arms according to miRBase (39) are indicated with arrows. (**B**) Sequences of miR-CLIP probes for miR-124-3p and miR-132-3p; cytidines labeled with biotin are indicated with X; adenosines labeled with psoralen are indicated with Y. (**C** and**D**) Luciferase reporter gene suppression by wild-type pre-miR-124 and pre-miR-132, and two miR-CLIP probes for each miRNA; HEK293T cells were co-transfected with luciferase reporter plasmids containing one reverse complementary target site against the respective miRNA and three concentrations of pre-miRNAs or miR-CLIP probes (0, 2.5, 10, 40 nM); *N* = 3. (**E** and **F**) Transcript levels of literature-reported miR-124 (E) and miR-132 (F) targets after transfection with 40 nM of the indicated RNA. Transcript levels were compared to transfection with negative control RNA, siCon (40); *N* = 3. Error bars indicate standard deviations. Asterisks denote statistical significance compared to 0 nM dose (C, D) or siCon treatment (E, F) assessed by two-way ANOVA Dunnett test whereas: ns *P* > 0.05, * *P* ≤ 0.05, ** *P* ≤ 0.01, *** *P* ≤ 0.001, **** *P* ≤ 0.0001.

There are several challenges in the design of a well-functioning miR-CLIP probe. The functional group and its linker should be positioned in pre-miRNA so that it does not interfere with Dicer processing and RISC loading/function (21,28,29). Also, although psoralen and its derivatives (i.e. trioxsalen) are considered as classical RNA–RNA cross-linking reagents, cross-linking is heterogeneous, is restricted mostly to uracil and is linker/sequence/structure-dependent (30). It has proven challenging to find ways to optimize these limitations. Therefore, we synthesized two miR-CLIP probes for each 3p miRNA, mindful that in contrast to the original miR-CLIP reagent miR-106a-5p, subsequent Dicer-processing would produce isomiRs with the psoralen located at distinct positions (Figure 1A,B; Supplementary Figure S1 and Supplementary Table S1). We introduced trioxsalen (hereafter, denoted psoralen or pso) at two sites in the seed of miR-132 (hp-132-1; hp-132-2), so as to cross-link with base-paired uracils in mRNA targets. For miR-124, we also placed a psoralen in the seed region (hp-124-3). In hp-124-1 we moved the psoralen outside of the miRNA seed region, toward the 3′-end of the miRNA so as to cross-link with unpaired uracils in target mRNAs.

In order to ensure that the pre-miRNA probes would enter the miRISC pathway and be processed as a native pre-miRNA, we transfected probes into HEK293T cells and assayed their activity in reporter assays using plasmids expressing *Renilla* luciferase mRNA, additionally containing a target site for the miRNA in its 3′UTR (Supplementary Table S2). While the miR-CLIP probes were approximately 1.5- to 2-fold less inhibitory than wild-type pre-miRNAs, robust reporter inhibition confirmed that the 3p guide strand of the probes is excised and forms an active miRISC (miR-124: Figure 1C; miR-132: Figure 1D).

We also examined probe activities against six literature-reported targets (miR-124-3p: *SP1* (31), *PTBP1* (32), *ROCK2* (33), *EZH2* (33), *VAMP3* (34) and *CTDSP1* (34); miR-132-3p: *PTEN* (35), *RB1* (36), *SIRT1* (37), *NAB1* (12), *MECP2* (38) and *EP300* (38); Supplementary Table S3). Wild-type pre-miR-124, hp-124-1 and hp-124-3 showed similar potencies, inhibiting all targets except *ROCK2* and *EZH2* to varying degrees (Figure 1D). Pre-miR-132 and hp-132-2 showed similar levels of target repression; however, hp-132-1 was barely active on the literature targets and was therefore not further investigated (Figure 1F). The results confirmed that the probes were processed as miRNA mimics to suppress their natural targets as part of RISC.

### The miR-CLIP protocol

**Figure 2.**
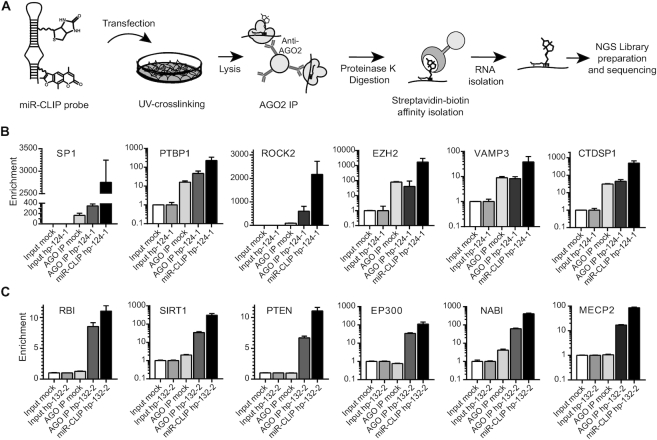
The miR-CLIP protocol. (**A**) Principal steps in the protocol (adapted from Imig *et al.* (21). (**B** and**C**) Enrichments of control targets after AGO2 IP (AGO IP) and in combination with streptavidin-biotin pulldown (miR-CLIP) were assessed using RT-qPCR for hp-124-1 (B) and hp-132-2 (C). Cycle-threshold (*C*t) values after AGO2 IP or after streptavidin-biotin pulldown were normalized to GAPDH levels and to expression in the respective input samples. Graphs show results of one replicate (see Supplementary Figure S2 for replicates). Log10 scale used for *PTBP1*, *EZH2*, *VAMP3*, *CTDSP1*, *SIRT1*, *EP300*, *NAB1* and *MECP2*.

HEK293T cells were selected for miR-CLIP experiments since they do not express miR-124 and miR-132, and therefore their targets were expected to be present at sufficiently high levels for robust capture, as rationalized in early efforts to identify miRNA targetomes (34,41). HEK293T cells were transfected with low concentrations of miR-CLIP probes and then briefly irradiated at 254 and 365 nm (Figure 2A). Cells were lysed and miRISC complexes were collected by IP with an anti-AGO2 antibody. Proteins were then degraded and RNA complexes were isolated on streptavidin beads, thereby enriching for targets of the miRNA probe. Various RNA samples were collected, ready for processing into libraries for sequencing: *Input mock* (RNA from mock-transfected cells), *Input probe* (RNA from probe-transfected cells), *AGO IP mock* (immunoprecipitated RNA from mock-transfected cells), *AGO IP probe* (immunoprecipitated RNA from probe-transfected cells) and *miR-CLIP probe* (immunoprecipitated RNA from probe-transfected cells, enriched on streptavidin-beads). The AGO2 IP step in the mock- and probe-treated samples, captures all transcripts and miRNAs present in RISC; the streptavidin step further enriches for transcripts that are cross-linked to the biotinylated miR-CLIP probe.

We performed control experiments using the aforementioned six targets of each miRNA to ensure the quality of the libraries prior to sequencing. Hence, we isolated aliquots of RNA from cells treated with hp-124-1, hp-124-3 and hp-132-2 and quantified the mRNAs using qPCR. As expected, we observed that levels of these targets increased from input through AGO IP, with the highest level of enrichment found in most cases in miR-CLIP samples (hp-124-1: Figure 2B; hp-132-2: Figure 2C, Supplementary Figure S2). Therefore, we proceeded to sequence two libraries from cells treated with hp-124-1, four libraries with hp-124-3 and three libraries with hp-132-2 (Supplementary Tables S4 and S5). For all libraries, we sequenced 11.9–40.9 million reads in total with a mapping rate from 52.6% to 90.1%, and 33.1% to 60.6% assigned to transcripts (Supplementary Tables S4 and S5). Pleasingly, the sequence analysis revealed that the great majority of assigned reads in the miR-CLIP libraries derived from the three probes, were assigned to protein-coding transcripts, consistent with a properly functioning miR-CLIP protocol (Supplementary Figure S3). Next, we searched the targetomes for putative target sites for miR-132 and miR-124 *k*-mers (6mer, 7mer, 8mer), both across whole transcripts and in 3′UTRs, using the sequence of miR-122 as a negative control. We defined a miRNA ‘targetome’ as the top 1000 transcripts enriched in the miR-CLIP samples compared to corresponding input samples (positive log-fold changes ranked by the *P*-value from edgeR).

The strongest and most inhibitory miRNA–mRNA interactions are reportedly the 8mer site (complementarity of miRNA nt 2–8, with adenosine opposite to nt 1), then the 7mer-M8 (7m8; nt 2–8) and 7mer-A1 sites (nt 2–7, with adenosine opposite to nt 1), followed by weaker 6mer base-pairing interactions from nt 2–7 or nt 3–8. In canonical interactions, the 5′ terminal nucleotide of the miRNA does not actually base-pair with the opposite nucleotide of the target strand—often an adenosine (42)—which is docked into AGO2. However, miR-124 and miR-132 begin with uridine, and thus sequence alignments between these miRNAs and their 7merA1 or 8mer targets formally extend to nt 1 (hereafter denoted guide-1 or g1 position) of the miRNA. Indeed, for miR-132, we found that 6mer, 7mer and 8mer sites beginning at positions g1 or g2 (U_1_A_2_A_3_C_4_A_5_G_6_U_7_C_8_) were highly enriched over expected occurrences in top transcript 3′UTRs (Supplementary Figure S4). For miR-124, enrichments for the same seed motifs were also found, though less prominently than for miR-132 (Supplementary Figure S5). Importantly, motifs corresponding to the seed of miR-122 (UGGAGUGU; negative control) were not enriched in either targetomes (Supplementary Figures S4 and S5). Taking these findings, together with the almost exclusive capture of protein-coding transcripts by miR-124 and miR-132, confirmed that miR-CLIP had captured selectively large numbers of *bona fide* canonical targets.

### miR-CLIP identifies new targets of miR-124 and miR-132

**Figure 3.**
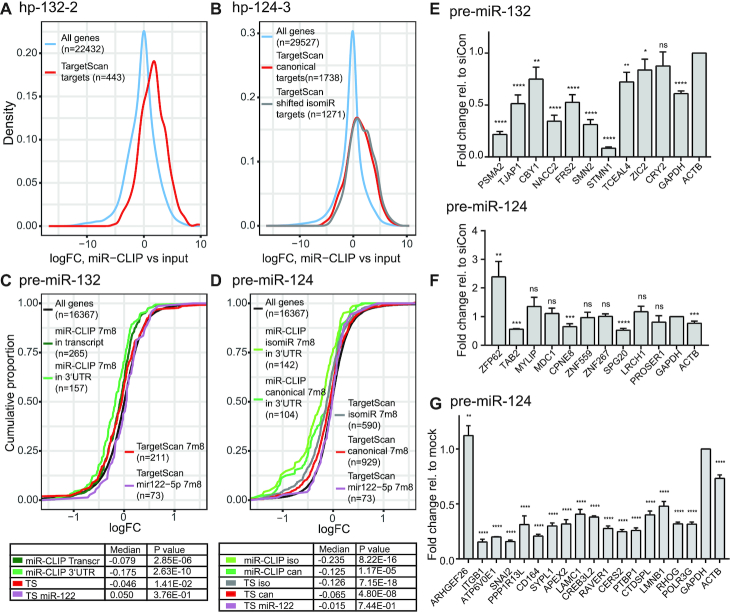
MiR-CLIP-targetomes of miR-124-3p and miR-132-3p. (**A** and **B**) Density plots of log2 fold changes of TargetScan-predicted targets in the hp-132-2 (A) and hp-124-3 (B) miR-CLIP samples, compared to the respective input samples (hp-132-2: *P* = 2.27e-61; hp-124-3: *P* = 8.47e-174 (miR-124), *P* = 9.58e-192 (*iso*-miR-124); Wilcoxon rank-sum test comparing gene subset to genes outside subset). (**C** and**D**) Cumulative distributions of log2 fold changes (logFC) in mRNA abundances after 40 nM transfections of pre-miR-132 (C) and pre-miR-124 (D) in HEK293T cells. The distribution of fold changes in different subsets of genes are plotted for miR-132- and miR-124-TargetScan-predicted targets with 7m8 sites in the top 1000 miR-CLIP (at least one 7m8 target motif in the 3′UTR or within the whole transcript); TargetScan-predicted miR-122-5p 7m8 seed targets were used as negative controls. *P* values obtained by Wilcoxon rank-sum test were obtained by comparing gene subsets to genes outside the subset. (**E**) Levels of transcripts in the top ten hp-132-2-captured targets assayed with qPCR after 40 nM pre-miR-132 transfection. (**F**) Levels of transcripts in the top ten hp-124-3-captured targets validated by qPCR after 40 nM pre-miR-124 transfection. (**G**) Transcript levels of 17 miR-124 strongly destabilized targets after transfecting 40 nM pre-miR-124 to the cells (cut-off at logFC <-0.8 (FC <0.57)). Significance compared to mock treatment. For (E, F) *N* = 4, (G) *N* = 3, error bars represent standard deviations. Asterisks denote statistical significance compared to siCon or mock respectively (Student’s *t* test): ns = *P* ≥ 0.05 * = *P* < 0.05, ** = *P* ≤ 0.01, *** = *P* ≤ 0.001, **** = *P* ≤ 0.0001.

We adopted two methods in order to validate indirectly the data from the miR-CLIP experiments. According to data in miRBase (39) and literature (23), *hsa-miR-124* produces two main miRNA isoforms: miR-124-3p.1 (miR-124) and miR-124-3p.2 (*iso*-miR-124), whereas *hsa-miR-132* yields one 3p miRNA (Figure 1A). First, we assessed the behavior of selected sets of miR-124- and miR-132-targets predicted by TargetScan (version 7.2) (12) in miR-CLIP libraries. We found that predicted canonical targets of miR-132 (443 mRNAs; Figure 3A), miR-124 (1738 mRNAs; Figure 3B) and *iso*-miR-124 (1271 mRNAs; Figure 3B) were present at significantly higher enrichments in miR-CLIP-samples, over the respective inputs, than non-target genes (Supplementary Figure S6). A similar response was obtained for targets of the miRNAs predicted by a second tool – miRTarBase, in which literature-reported miRNA–target interactions are annotated (43).

Second, we demonstrated previously with miR-106a (21) that predicted targetomes and experimentally captured (miR-CLIP) targetomes of a miRNA can be compared in a functional context after measuring the transcriptomic response of cells to the exogenously delivered miRNA mimic. Hence, we treated cells with wild-type pre-miR-124 or pre-miR-132 and performed sequencing on isolated RNA. Predicted targets of the negative control miR-122 (canonical 7m8 targets; *n* = 73;) were unaffected by the pre-miRNA transfections. In contrast, 211 mRNAs that are expressed and are predicted by TargetScan to carry a 7m8 site for miR-132 in their 3′UTRs were suppressed as a group with high significance (Figure 3C). In the top 1000 miR-CLIP-targets we identified 157 and 265 motifs complementary to nt 2–7 of miR-132 in the 3′UTR or anywhere in the transcript, respectively; the mRNAs were suppressed by a greater degree than the aforementioned 211 predicted targets (Figure 3C). A similar outcome was observed for miR-132 targets present in miRTarBase (Supplementary Figure S7).

Next, we assayed the effects of pre-miR-132 transfection on the top ten miR-CLIP-captured targets using RT-qPCR. Nine from ten targets were suppressed (Figure 3E); from which seven bore TargetScan-predicted sites for miR-132-3p (including *GAPDH*). None of the remaining targets (*CBY1*, *SMN2*, *ZIC2*) were predicted as targets, though *ZIC2*—a transcription factor important in brain development (44)—has been isolated previously using a CLIP protocol (45).

Similar findings were noted for miR-124; 929 and 590 transcripts that are expressed and predicted to carry TargetScan 7m8 motifs in their 3′UTRs for miR-124 and to *iso*-miR-124 (Supplementary Tables S6 and S7), respectively, were suppressed (Figure 3D and Supplementary Figure S7). However, 104 and 143 miR-CLIP-captured targets with complementary motifs to nt 2–7 of miR-124 and *iso*-miR-124 were repressed even more strongly. Once again, we examined the response of selected miR-CLIP-targets on mRNA and protein (*vide infra*) after transfection of wild-type pre-miR-124 into HEK293T cells (Figure 3F, G). The levels of four from the top ten targets (Figure 3F) were decreased, all of which bore 7m8 sites to *iso*-miR-124, and in some cases, to both isoforms. These included *ACTB*, which carries one conserved binding site for *iso*-miR-124; indeed, *ACTB* protein was also suppressed selectively at the protein level by *iso*-miR-124, but not miR-124 (*vide infra*).

The cumulative fold-changes representation of the miR-124 targetome after pre-miR-124-transfection revealed a subset of 17 targets that were unusually highly repressed (Figure 3D). The inhibition of 16/17 targets was successfully confirmed by qPCR (Figure 3G), and several of these were repressed by miR-124 and/or *iso*-miR-124 at the protein level by LC-mass spectrometry analysis (*vide infra*). Eleven of them code for proteins whose mean expressions are fully or partially depleted in brain (Supplementary Table S8 (https://www.proteinatlas.org/)), in line with a high expression of miR-124 in cells of the brain.

**Figure 4.**
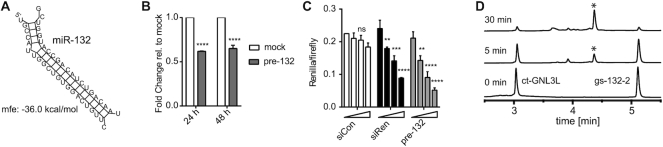
GNL3L is regulated by miR-132-3p. (**A**) RNAhybrid calculated binding of miR-132-3p to a predicted target site in the GNL3L 3′UTR (figure retrieved from RNAhybrid on 11 September 2018). (**B**) Fold change of GNL3L mRNA levels 24 and 48 h after 40 nM pre-miR-132 transfection. *N* = 2 (significance compared to mock treatment calculated by Sidak test whereas: ns (*P* > 0.05), * (*P* ≤ 0.05), ** (*P* ≤ 0.01), *** (*P* ≤ 0.001), **** (*P* ≤ 0.0001)). (**C**) Luciferase assay with psiCHECK2 plasmid containing the target site shown in (A) in the 3′UTR of the *Renilla* luciferase; *N* = 2. Significance compared to 0 nM dose calculated by two-way ANOVA Dunnett test: ns (*P* > 0.05), * (*P* ≤ 0.05), ** (*P* ≤ 0.01), *** (*P* ≤ 0.001), **** (*P* ≤0.0001). (**D**) *In vitro* photo-crosslinking of psoralen-modified miR-132 guide (gs-132-2) to a 15-nt counterstrand (ct-GNL3L) bearing the putative GNL3L target site; HPLC chromatograms of the annealed duplex before and after 5 and 30 min of irradiation at 365 nm; * indicates position in the chromatogram of the cross-linked duplex (mass calc.: 12107.6, mass found: 12107.2).

Hp-132-2 captured the mRNA of guanine nucleotide-binding protein-like 3-like protein (*GNL3L*) (Figure 4), a nucleolar GTPase whose depletion is associated with G2/M arrest in the cell cycle (46,47). TargetScan predicts a huge number of poorly conserved putative target sites on GNL3L among vertebrates. Therefore, normally GNL3L would not be considered as a high confidence target of miR-132. We identified a putative target site in its 3′UTR with unusually high complementarity to miR-132 (Figure 4A), that was somewhat reminiscent of the previously described miR-196-HOXB8 interaction (48). The binding and regulation of *GNL3L* by miR-132 was confirmed in two cellular assays and in an *in vitro* assay, which is consistent with its capture by miR-CLIP. Hence, pre-miR-132 was transfected into HEK293T cells; GNL3L mRNA was assayed using qPCR and showed robust suppression after 24 and 48 h (Figure 4B). To confirm that this regulation indeed derived from the putative 3′UTR site (Figure 4A), a luciferase reporter plasmid was prepared, containing that site. Next, the reporter plasmid was co-transfected with pre-miR-132, into HEK293T cells resulting in the inhibition of luciferase signal, with similar efficiency to the siRNA positive control (Figure 4C).

In order to confirm the ability of hp-132-2 to cross-link with GNL3L in cells, we examined its reaction under model conditions *in vitro*. A simplified analogue of hp-132-2 (gs-132-2; Supplementary Table S1 and Supplementary Figure S8) was synthesized and incubated with a short RNA of identical sequence to the presumed GNL3L target site (ct-GNL3L9; Supplementary Table S1) and irradiated at 365 nm. Cross-linking of the model miRNA to its target was followed by HPLC/LC-MS. One new product with the expected mass for cross-linking was visible in the chromatogram already after 5 min, which is consistent with the capture of GNL3L in cells by hp-132-2 (Figure 4D).

One objective of this study was to identify any dependence of a miR-CLIP targetome on the site-specific location of the psoralen groups in the probes. Complementarity in a miRNA–mRNA interaction is typically most extensive in the seed. Thus, the psoralen in hp-124-3 was expected to cross-link to target uridines that base-pair with g2 or g3; whereas, in hp-124-1 reactive uridines close to the psoralen at the 3′ end of the probe would probably not be base-paired with the miRNA (Figure 1A). The targetomes from hp-124-1 and hp-124-3 showed an approximately 50% overlap in the top 1000 captured targets (Supplementary Tables S6, S7 and Supplementary Figure S9), which increased to 61–62% when the target pool was limited to the top 600 or 200 transcripts. Nevertheless, we cannot exclude that the capture of weaker miRNA–mRNA interactions may have been at least partly dependent on positioning of the modifications, affecting the efficiency of cross-linking and/or sequence dependence of psoralen-reactivity. Although the analysis was performed only on two miR-CLIP probes, the results reinforced the notion that a thorough understanding of psoralen reactivity at nucleobases might aid the design of improved miR-CLIP probes.

### miR-124 isoforms regulate common targets to different degrees

**Figure 5.**
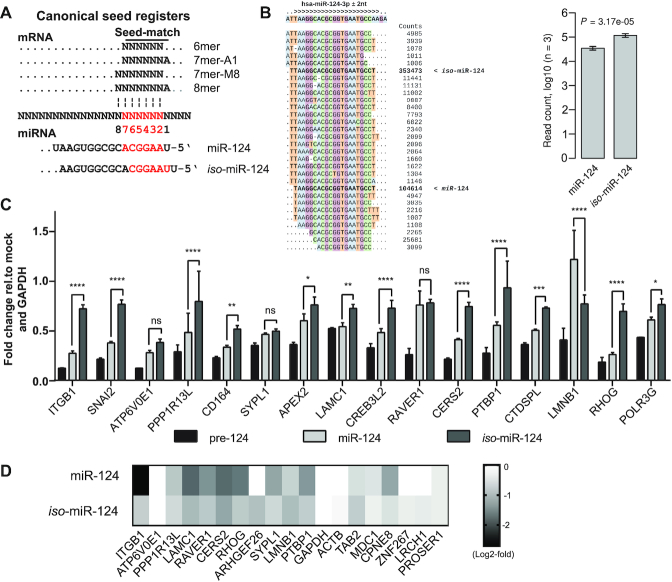
Pre-miR-124 produces isomiRs with distinct targeting properties. (**A**) MiRNA–mRNA binding motifs via interactions involving nucleotides g1-g8 of miR-124 and *iso*-miR-124 (adapted from(2)). (**B**) Small RNA-Seq read alignments to *hsa-miR-124* (left) and log10-transformed counts of small RNA-Seq reads, supporting miR-124 and iso-miR-124 in libraries extracted from HEK293T cells transfected with 40 nM pre-miR-124 (right). The indicated *P* value was calculated with a two-tailed, paired *t*-test (see Supplementary Figure S10; for clarity, nucleotides are color-coded). (**C**) Fold change of miR-124 target mRNAs after treating HEK293T cells with 40 nM of pre-miR124, miR-124 or *iso-*miR-124 duplexes. Asterisks denote significance between miR-124 and *iso-*miR-124 treatment. Significance calculated by two-way ANOVA Dunnett test: ns (*P* > 0.05), * (*P* ≤ 0.05), ** (*P* ≤ 0.01), *** (*P* ≤ 0.001), **** (*P* ≤0.0001). (**D**) Inhibition of selected miR-CLIP targets determined by multiplexed protein identification and quantitative analysis by tandem mass spectrometry. Heatmap represents the fold change of the designated targets in miR-124 or iso-miR-124 transfected cells with respect to mock transfected cells. Average fold change is computed from three independently transfected biological replicates of each treatment.

*hsa-miR-124-1* produces two main 3p miRNAs that differ by one nucleotide at their 5′-ends (Figures 1A and 5A). Analysis of sequencing reads from pre-miR-124-transfected HEK293T cells showed that *iso*-miR-124 was 3.4-fold more prevalent than miR-124 (Figure 5B and Supplementary Figure S10). This contrasts with data from miRBase (39) and data from human retinal cells (26) in which miR-124 was the dominant isoform, and it adds to the evidence, that production of isomiRs is cell-type dependent (49). This distribution was consistent with capture of larger numbers of transcripts carrying 7m8-target sites by *iso*-miR-124 than by miR-124 (142 and 104 transcripts, respectively) (Supplementary Figure S11). Furthermore, it was consistent with the outcome of pre-miR-124 transfections that yielded greater repression of miR-CLIP transcripts bearing a 7m8 target motif from *iso*-miR-124 than from miR-124 (median logFC = -0.235 and -0.125, respectively) (Figure 3D).

A small number of works have addressed the individual targeting properties of isomiRs. Karali *et al.* showed that *iso*-miR-124 suppresses a target site from *CDH11* (26) that is mostly unaffected by miR-124; Llorens *et al.* were unable to show differences between miR-101 and its isomiR on a variety of targets (50); but Tan *et al.* identified distinct targets of miR-9 and *iso*-miR-9 (49). From a genome-wide study, Cloonan *et al.* postulate that isomiR populations evolve to target common mRNAs (51).

In order to provide insight on this aspect of miRNA function, we studied the effects of individual miR-124 isoforms at RNA and protein levels in HEK293T cells on the same 16 mRNAs strongly inhibited by pre-miR-124 (Figure 3G) using duplexes of miR-124 and *iso*-miR-124 (Supplementary Table S9). Silencing activities of the mimics were confirmed using a luciferase reporter construct (not shown). Most of the UTRs in these targets carry predicted target sites for both miR-124 isoforms, and therefore it was not possible to dissect the activity of any putative individual miRNA–mRNA interaction in the native mRNAs (Supplementary Table S10). Interestingly, pre-miR-124 repressed the 16 mRNAs to a much greater extent than individual treatments with the two isomiRs (Figure 5C). This effect was consistent with synergistic repression of a target by two isomiRs produced from a common pre-miR-124 precursor. However, we cannot rule out that it may have been at least partly due to Dicer-assisted AGO-loading of the pre-miRNA (52), or possibly a technical artefact of the experiment, e.g. variable transfection efficiencies. For 12/16 mRNAs, miR-124 was more active than *iso*-miR-124; for three mRNAs (*SYPL1*, *ATP6V0E1*, *RAVER1*), the miRNA isoforms were equipotent, and for one (*LMNB1*) *iso*-miR-124 was more active.

We used multiplexed protein identification and quantitative analysis by tandem mass spectrometry (MS) to examine the response of 19 aforementioned validated miR-CLIP targets (Figures 3F,G and 5C) to miRNA transfections. In total, 17/19 targets were suppressed by miR-124 and/or *iso*-miR-124. In 8/10 cases, inhibition at the protein level followed the same pattern as at the mRNA level (Figure 5D). *Iso*-miR-124 was more potent than miR-124 on 6/17 targets (*LMNB1*, *TAB2*, *LRCH1*, *ZNF267*, *ACTB*, *ARHGEF26*). Overall, validation of targets at mRNA and protein levels was at a very high level, speaking to the quality of the miR-CLIP experiment.

The g1 position of miR-124 is equivalent to g2 of *iso*-miR-124, and it follows that g7 of miR-124 is equivalent to g8 of *iso*-miR-124 (Figure 5A). Thus, an 8mer site in any given miR-124 target represents a 7m8 site or an 8mer site for *iso*-miR-124, depending on the nucleotides opposite g1 and g2 (Figure 5A). Thus, a strong regulation of such sites by both isoforms can be expected. Indeed, *APEX2*, which has an 8mer site for *iso*-miR-124 and miR-124 in its 3′UTR (Supplementary Figure S12), was inhibited similarly by the two isoforms (Figure 5C). On the other hand, isoform-selective targeting is likely where base-pairing to the target does not extend beyond 7 nt of the seed in one isoform. For example, *LMNB1* has a predicted 7merA1 site in its 3′UTR for *iso*-miR-124, which represents a weakly active 6mer site for miR-124 (Supplementary Figure S12), consistent with the greater suppression by the former on mRNA and protein levels (Figure 5C,D). In summary, the data from these 16 targets confirmed that miR-124 isoforms may have individual targets, but most of this group were inhibited by both miR-124 and *iso*-miR-124.

### Isoforms of miR-124 regulate G-bulged targets specifically

**Figure 6.**
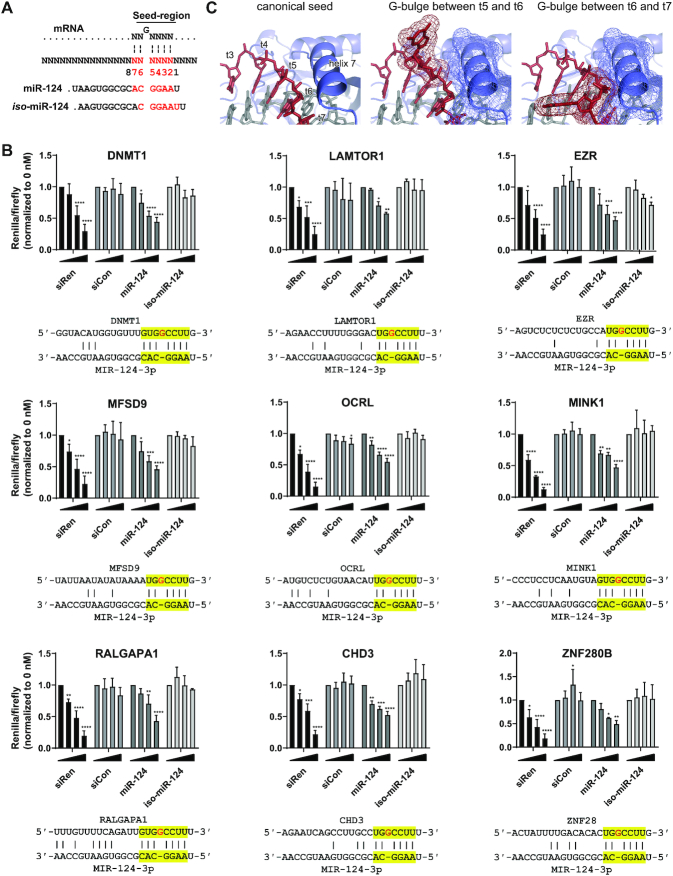
Regulation of G-bulged target sites by miR-124. (**A**) Alignments of the seed registers from miR-124 and *iso*-miR-124 with a G-bulge binding site (for insert sequence see Supplementary Table S4). (**B**) Luciferase reporter assay of constructs bearing putative G-bulge target sites. Reporters carrying an indicated site (sequences below bar-graphs) were co-transfected, with 0, 2.5, 10 or 40 nM, of positive control anti-Renilla siRNA (siRen), negative control siCon, miR-124 or *iso*-miR-124. Luciferase activity represents Renilla/firefly values normalized to 0 nM and is expressed as mean ± S.D. Asterisks denote significance between miR-124 and *iso*-miR-124 treatment at the same concentrations; *N* = 3. Significance calculated by two-way ANOVA Dunnett test whereas: ns (*P* > 0.05), * (*P* ≤ 0.05), ** (*P* ≤ 0.01), *** (*P* ≤ 0.001), **** (*P* ≤0.0001). (**C**) Models of AGO2-miR-124 and *-iso*-miR-124 complexes with a G-bulge target: detail of the seed-region of AGO2:miR-124-3p bound to a fully complementary target (based on PDBID 4w5t) (left); AGO2 in blue, miR-124-3p in gray, target in red; positions of nucleotides t3-t7 and of helix 7 are indicated. Detail of AGO2:miR-124-3p bound to a target with a G-bulge between positions opposite g5 and g6 (based on PDBID 4w5t) (middle); helix 7 and the flipped G are shown as a mesh representation. Detail of AGO2:isomiR-124-3p bound to the same G-bulged target, with the bulge now shifted to between positions opposite g6 and g7 (based on PDBID 4w5r) (right).

A recent AGO-HITS CLIP study identified numerous targets of miR-124 featuring a G-bulge targeting pattern, in which a guanosine nucleotide in the mRNA opposite seed positions 5–6 is bulged out (Figure 6A) (23). We searched for similar target sites in the top 1000 transcripts of hp-124-1 and hp-124-3 targetomes, finding 94 and 91, respectively, of which 44 transcripts were shared (Supplementary Figure S11a). Thirty-five of these 44 mRNAs were excluded from further analysis because they carried additional predicted canonical target sites that would confound investigation of the G-bulge motifs. The *MINK1* mRNA, which was identified in the original study (23), was present in the remaining nine targets, once again speaking to the robustness of the miR-CLIP method. Levels of the nine mRNAs were mostly unaffected by pre-miR-124 transfections (Supplementary Figure S13), suggesting that these particular sites do not undergo mRNA destabilization upon miRNA binding, which is consistent with results from three high-throughput studies (10,12,24). The nine G-bulge motifs were therefore cloned into dual luciferase reporter plasmids. Pleasingly, readout from the co-transfection of the respective reporters and pre-miR-124 into HEK293T cells, demonstrated that all nine predicted G-bulge target sites (*DNMT1, LAMTOR1, EZR, MFSD9, OCRL, MINK1, RALGAPA1, CHD3, ZNF280B*) repressed luciferase (Supplementary Figure S14), similarly to treatment with the siRNA control.

It has been proposed that regulation of a G-bulge site by miR-124 involves transient pairing of seed nucleotides 2–6 with the mRNA (Figure 6A). The duplex then adopts a more thermodynamically stable state with the bulged guanosine between positions 5 and 6 presumably stabilized by an interaction with AGO2 (23). Indeed, a recent, independent analysis of binding kinetics and AGO2 cleavage rates for siRNAs and miRNAs, has described how an extra guanosine opposite g5-g6 of two other miRNAs (miR-21, let-7) creates a similarly favorable effect (24). We reasoned that given tight interactions between AGO2 and the miRNA, particularly close to helix 7 (53), that the shifted seed of *iso*-miR-124 would be unable to force these conformational changes at positions g6-g7 in order to engage G-bulge targets.

We therefore tested the nine reporter genes for inhibition by miR-124 and *iso*-miR-124 in HEK293T cells (Figure 6B, see also Supplementary Figure S15). While miR-124 inhibited the expression of all nine G-bulged sites, *iso*-miR-124 showed no statistically significant activity at all. Finally, we examined whether this selective inhibition by miR-124 isoforms could be observed on the endogenous proteins. We transfected HEK293T with both isoforms and isolated total protein for a targeted-proteomics analysis. Of the nine proteins, DNMT1, LAMTOR1 and ZNF280B were detectably expressed in the cells and their levels were significantly reduced by miR-124 by 31%, 38% and 36%, respectively (Supplementary Table S11); they were not suppressed by isomiR-124. Overall, these data were consistent with our structural hypothesis in which the isomiR is unable to adopt the correct conformation to engage the target site. To test our hypothesis, we used crystal structures of AGO2:miRNA:guide complexes (42) to model the binding of miR-124-3p with a canonical target, and the binding of miR-124-3p and isomiR-124-3p with a G-bulged target. The seed region is in close proximity to helix 7 (Figure 6C), which has been reported to facilitate binding and release of miRNA targets (53). When we modelled a flipped-out G-bulge between positions t5 and t6, we noted a slight distortion of the backbone, but no apparent clashes with helix 7, nor with other nearby regions of AGO2 (Figure 6C). We then attempted to model the binding of *iso*-miR-124-3p for an analogous G-bulge, which resulted in the bulge now being positioned between nucleotides t6 and t7. While our model was limited by the use of a rigid AGO2, the distortions imparted to the RNA backbone resulted in a clash with helix 7 (Figure 6C). Our model would thus support the hypothesis that the presence of a G-bulge might assist selective targeting of mRNAs by 5′ miRNA isoforms, in this case a specific targeting by miR-124 but not its isomiR.

## DISCUSSION

The scope and importance of non-canonical miRNA functions as a class is difficult to ascertain due to a paucity of rigorous methods to identify all of the targets of a miRNA in a cell. The best target prediction programs function with precisely defined seed sequences. Therefore, they work less effectively for interactions that involve partial seed-target complementarity, or for miRNA precursors that are processed heterogeneously. Similarly, current CLIP methods are unsatisfactory since it is thought that they capture mostly transient, low-affinity (24), non-functional (12) interactions. In addition, identification of the precise miRNA (family member, isomiR) implicated in the interaction is usually not possible.

MiR-CLIP pre-miRNA probes are ‘masked’ reagents that are processed in cells by Dicer into functional miRNA mimics bearing cross-linker groups. Previously, we used miR-CLIP to identify the targetome of miR-106a, which included canonical and non-canonical targets alike. In this study, we extended the miR-CLIP technique to 3p miRNAs, using miR-132 and miR-124 as examples. In contrast to the original study on a 5p-miRNA, Dicer-processing of the two pre-miRNA probes in this study produced isomiRs with the cross-linker located at different seed positions. This increased the complexity of the experiment, the data analysis and the follow-up studies. The miR-CLIP experiments identified and confirmed dozens of miR-132 and -124 targets, which were almost exclusively protein-coding mRNAs (Supplementary Figure S3). We observed substantial overlap between targetomes determined from the use of two different probe designs with psoralen and biotin at distinct sites in the pre-miRNA (Supplementary Figure S9). This bodes well for the widescale use of miR-CLIP with any miRNA.

Sequencing small RNAs from cells and tissues indicates that Dicer cleavage of a pre-miRNA on the 3p arm—under the influence of TRBP (54) and other RBPs—is heterogeneous and cell type-dependent (26,49,50). This yields a population of isomiRs with distinct seed sequences, and therefore a potentially expanded range of targeting. The net functional output from a miRNA gene is assumed to be the sum activities from individual isomiRs (51), albeit influenced by various parameters, e.g. sequence, concentration, potency in RISC etc. It is presently unclear how the presence of isomiRs expands the regulatory breadth of a miRNA’s activity; in some cases, prediction algorithms suggest a high degree of targeting overlap between isomiRs (e.g. miR-101 (50)); in others, they predict largely different targetomes (e.g. miR-9 (49)). Some have argued that generally, miRNAs and their isomiRs evolve to drive mRNA networks with similar biology and that their cooperative activity permits target repression with fewer off-target effects (51). However, this does not preclude target-specific activities of isomiRs, which has been shown experimentally for a few miRNAs having important tissue-selective functions (26,49).

In HEK293T cells, cleavage of pre-miR-124 produces mainly miR-124 and *iso*-miR-124 in an approximate 1:3 ratio (Figure 5B). Consistent with this, miR-CLIP data revealed subsets of mRNAs with predicted 7m8 target sites for both miR-124 and *iso*-miR-124 (Supplementary Figure S11), suggesting that the pre-miRNA probe was processed into functional miR-124- and *iso*-miR-124 mimics armed with psoralen groups. We studied the effects of transfecting into cells pre-miR-124, miR-124 and *iso*-miR-124 on a subset of 16 miR-CLIP targets, most of which carry 7mer and 8mer target motifs for both isomiRs. These were repressed in most cases more strongly by miR-124 than by *iso*-miR-124. However, LMNB1 mRNA was selectively inhibited by *iso*-miR-124, possibly due to a single base in the mRNA changing its seed-pairing from 7merA1 to a weaker six-nucleotide stretch for miR-124 (Supplementary Figure S12). Strikingly, pre-miR-124 was a far greater inhibitor of these 16 targets than the individual isomiRs, which aligns with the hypothesis of cooperative isomiR activity (51). As an aside, this finding may suggest that greater potency and optimal selectivity is available from pre-miRNA precursors than miRNA mimics in therapeutic settings.

Recent structural (55), biophysical (24) and biochemical studies (10) have provided key insights on novel types of miRNA–target interactions, both inside and outside the seed region. For example, relatively long looped-out target sequences are now known to be well tolerated in RISC and may be widespread and influential (24,55). MiR-CLIP-124 probes captured a large set of mRNAs with predicted seed-binding interactions to G-bulged sites. This non-canonical targeting motif was originally identified on the genomic scale in an Ago-HITS-CLIP study of miR-124 performed in mouse brain (23). The authors postulated that one G of a G_2_-dinucleotide of the target plays a transient stabilizing role for AGO2 binding in RISC. In the work, individual targets and target sites were not subject to follow-up studies at the mRNA and protein levels. Moreover, two later reports concluded that G-bulge interactions are likely not functional since no changes are seen on target RNA levels in cells treated with miR-124 or other miRNAs (12,24). MiR-CLIP captured dozens of targets containing this predicted binding motif, nine of which were prioritized for detailed follow up, including MINK1 which was identified in the original work (23). None of the mRNAs were affected by pre-miR-124 transfection. However, insertion of the predicted site into luciferase-expressing plasmids produced a set of nine reporters, all of which were strongly suppressed by pre-miR-124 and miR-124, but were completely resistant to *iso*-miR-124. For DNMT1, LAMTOR1 and ZNF280B, specific inhibition by miR-124 was confirmed on the endogenous proteins. These observations warn against assigning functionality to putative non-canonical miRNA–target interactions on the basis of changes in target mRNA levels (12,24,56). A recent structural study of AGO2 bound to a miRNA–mRNA target, provided a credible structural explanation for our findings, whereby a G-bulge precisely positioned in the target strand by AGO2 is favorable for binding to miR-124, but not to *iso*-miR-124 (Figure 6C). It is tempting to speculate that G-bulged targeting—stabilized or de-stabilized by an AGO2–target interaction—provides an effective means to differentiate the activity of two isomiRs that differ by a single 5′ nucleotide.

It is commonly accepted that most of the biology of a miRNA revolves around seed-based targeting (2,10). However, it seems increasingly likely that yet-unidentified, potentially widespread types of non-canonical miRNA–mRNA interactions await discovery. The miR-CLIP technique described here—with rigorous controls—reliably identifies the targetome of a defined miRNA sequence in cells. Here, we have extended miR-CLIP to the complexity of 3p miRNAs, showing the robustness of the chemistry with probes of different designs. We demonstrated that miR-CLIP provides high confidence starting points for follow-up investigations of novel miRNA biology and our study clarified a discordance in the literature about the functionality of G-bulged sites (12,23). In addition, we showed that miRNA isoforms of miR-124 have common and specific targets, and that the G-bulge targeting properties of miR-124 derives from one isomiR. In a broader context, these data show a rare example of how heterogeneous processing of miRNA precursors combined with a non-canonical seed-based targeting endows isomiRs with a distinct targeting profile.

## DATA AVAILABILITY

Raw data from hp-124-1- and hp-124-3-sequenced libraries (Supplementary Table S4) uploaded to ArrayExpress under accession number E-MTAB-8517.

Raw data from hp-132-2-sequenced libraries (Supplementary Table S5) uploaded to ArrayExpress under accession number E-MTAB-8517.

## Supplementary Material

gkaa1117_Supplemental_Files
