# Supplementary material for: MiR-CLIP reveals iso-miR selective regulation in the miR-124 targetome
Source: Nucleic Acids Res. 2020 Dec 9;49(1):25–37. doi: 10.1093/nar/gkaa1117 (PMC7797034; doi:10.1093/nar/gkaa1117)
Supplement: gkaa1117_Supplemental_Files [file gkaa1117_supplemental_files.zip › Wang_SI.pdf]

Supplementary Information

MiR-CLIP reveals *iso*-miR selective regulation in the miR-124 targetome

Yuluan Wang[1], Charlotte Soneson[2], Anna L. Malinowska[1], Artur Laski[1], Souvik Ghosh[3], Alexander Kanitz[3], Luca F.R. Gebert[4], Mark D. Robinson[2] and Jonathan Hall[1]

[1]Institute of Pharmaceutical Sciences, ETH Zurich, Vladimir-Prelog-Weg 4, CH-8093 Zurich.

Table of Contents

Supplementary Figures **2**

Supplementary Figure S1: Chromatograms of miR-CLIP probes and wild type controls 7
Supplementary Figure S2: miR-CLIP enrichments of all the miR-CLIP replicates sequenced. 10
Supplementary Figure S3: Classes of RNAs identified by RNA sequencing from miR-CLIP experiments. ... 11
Supplementary Figure S4: Reverse complementarity of the miR-CLIP-captured targets to k-mers present in miR-132-3p. 12
Supplementary Figure S5: Reverse complementarity of the miR-CLIP-captured targets to k-mers from miR-124-3p. 13
Supplementary Figure S6: Density plots of Targetscan-predicted and miRTarBase-reported targets from miR-CLIP samples. 14
Supplementary Figure S7: Cumulative distributions of target fold changes after pre-miRNA treatment of HEK293T cells. 15
Supplementary Figure S8: LC-MS chromatograms of reagents used for the *in vitro* cross-linking experiment. 16
Supplementary Figure S9: Overlap of datasets containing RNAs captured by hp-124-1 and hp-124-3. 18
Supplementary Figure S10: Small RNA-Seq read alignments to miR-124 (separate file) 19
Supplementary Figure S11: Overlapping datasets of target RNAs bearing selected seed target motifs, captured by the miR-124 miR-CLIP probes hp-124-1 and 124-3. 20
Supplementary Figure S12: Sequence alignments for predicted canonical targeting of LMNB1 and APEX2 by miR-124 and iso-miR-124. 21
Supplementary Figure S13: Transcript level of G-bulge targets upon miR-124 transfection determined by SYBR green RT-qPCR. 22
Supplementary Figure S14: Luciferase reporter assays for effect of pre-mir-124 on 9 discovered G-bulge target sites. 23
Supplementary Figure S15: Alignment of proposed binding of miR-124 and *iso*-miR-124 to DNMT1. 24

Supplementary Tables **25**

Supplementary Table S1: Characterization of synthesized oligoribonucleotides 25
Supplementary Table S2: Luciferase plasmid reporter inserts 26
Supplementary Table S3: qPCR primers used for SYBR green qPCR 27
Supplementary Table S4: QC parameters of hp-124-1- and hp-124-3-sequenced libraries 29
Supplementary Table S5: QC parameters of the hp-132-2-sequenced libraries 30
Supplementary Table S6: Hp-124-1 captured transcripts 31
Supplementary Table S7: Hp-124-3 captured transcripts 35
Supplementary Table S8: Properties of the top hp-124-3 captured, downregulated targets 39
Supplementary Table S9: Sequences of miR-124 duplexes 40
Supplementary Table S10: Properties of the top hp-124-3 captured, downregulated targets 41
Supplementary Table S11: Selective inhibition of proteins containing G-bulged sites by miR-124 (separate Excel file) 42

Supplementary Methods **43**

Scheme S1. Preparation of trioxsalen (psoralen)/biotin bis-labeled pre-miR-CLIP probes. 43
Chemical synthesis 43

Oligonucleotide synthesis, deprotection and purification 43
MiR-CLIP protocol 44
Small RNA sequencing 45
Sequencing analysis 45
Shotgun LC-MS experiments 45
Targeted PRM-LC-MS analysis 46

References 47

Supplementary Figures

LC-MS chromatogram of pre-miR-124

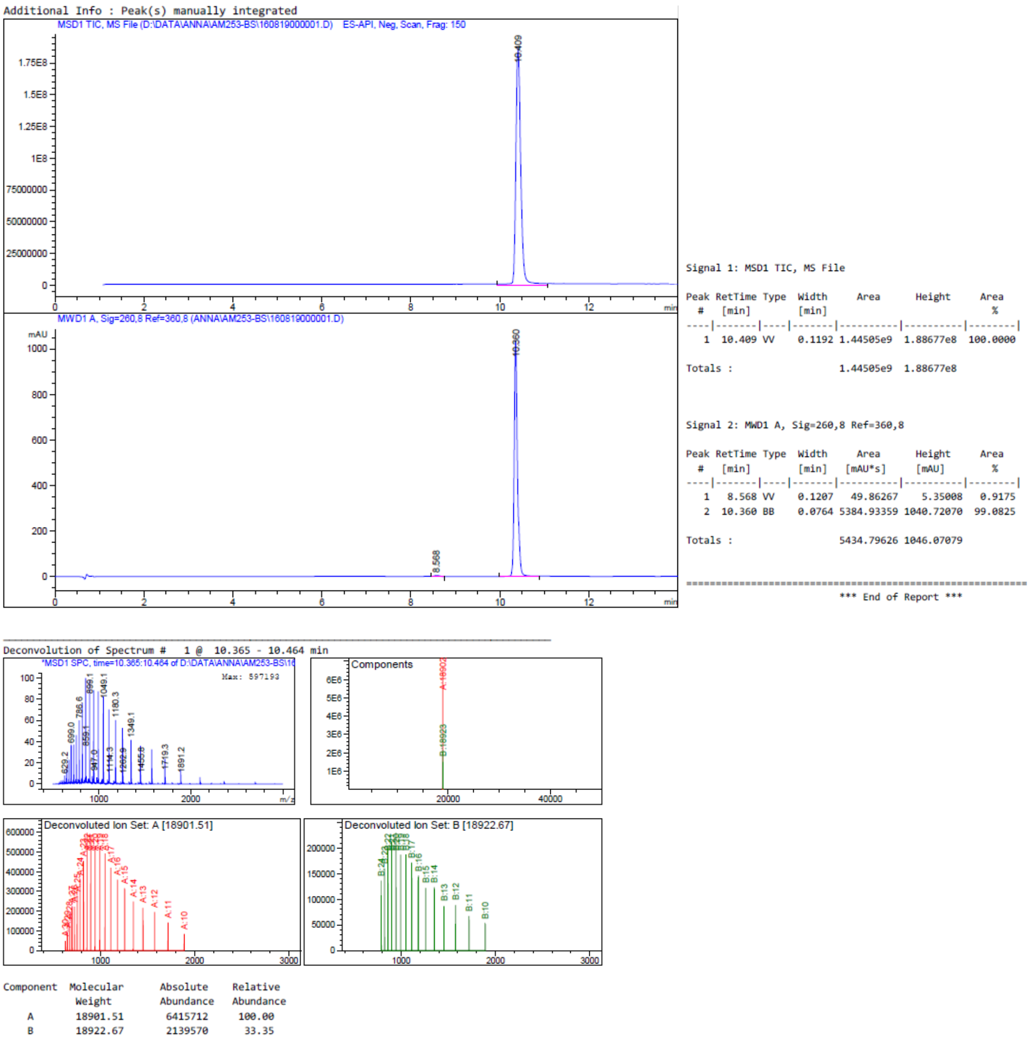

LC-MS chromatogram of hp-124-1

Additional Info : Peak(s) manually integrated

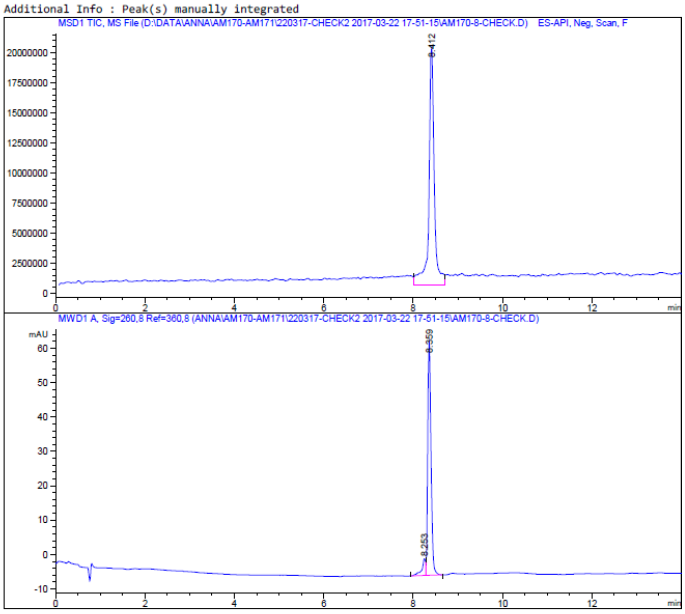

Signal 1: MSD1 TIC, MS File

Peak #	RetTime [min]	Type	Width [min]	Area	Height	Area %
1	8.412	VV	0.1171	1.64048e8	2.02114e7	100.0000
Totals :				1.64048e8	2.02114e7	

Signal 2: MWD1 A, Sig=260,8 Ref=360,8

Peak #	RetTime [min]	Type	Width [min]	Area [mAU*s]	Height [mAU]	Area %
1	8.253	BV	0.0822	28.68416	4.90342	7.7253
2	8.359	VB	0.0745	342.61847	68.35164	92.2747
Totals :				371.30263	73.25506	

Deconvolution of Spectrum # 1 @ 8.345 - 8.470 min

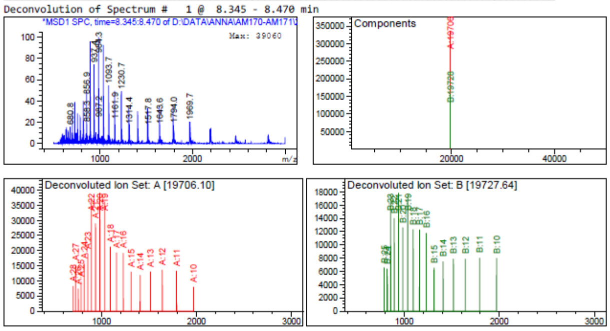

Component	Molecular Weight	Absolute Abundance	Relative Abundance
A	19706.10	336302	100.00
B	19727.64	155328	46.19

*** End of Report ***

LC-MS chromatogram of hp-124-3

Additional Info : Peak(s) manually integrated

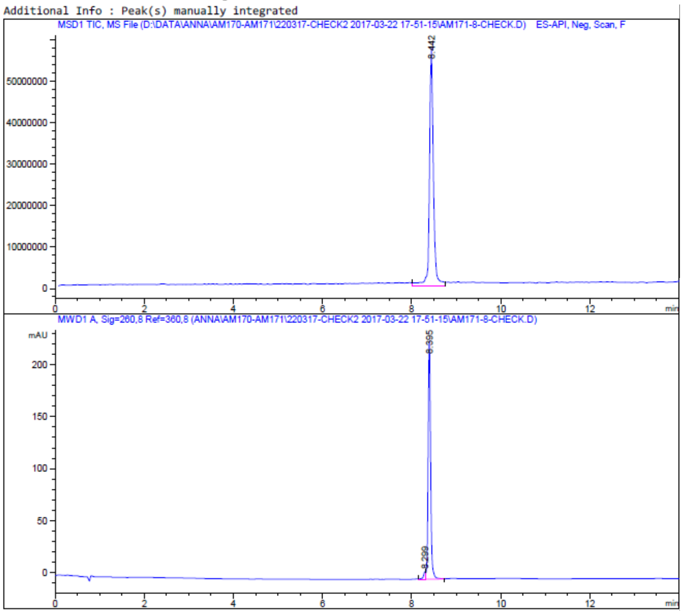

Signal 1: MSD1 TIC, MS File

Peak #	RetTime [min]	Type	Width [min]	Area	Height	Area %
1	8.442	VV	0.0931	3.50079e8	5.77698e7	100.0000

Totals : 3.50079e8 5.77698e7

Signal 2: MWD1 A, Sig=260,8 Ref=360,8

Peak #	RetTime [min]	Type	Width [min]	Area [mAU*s]	Height [mAU]	Area %
1	8.299	VV	0.0555	26.21266	7.02226	2.7685
2	8.395	VB	0.0629	920.62122	228.99434	97.2315

Totals : 946.83387 236.01660

Deconvolution of Spectrum # 1 @ 8.420 - 8.520 min

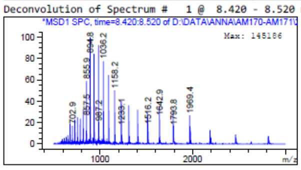

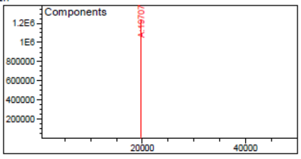

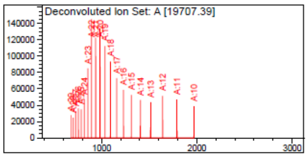

Component	Molecular Weight	Absolute Abundance	Relative Abundance
A	19707.39	1240225	100.00

*** End of Report ***

LC-MS chromatogram of pre-miR-132

Additional Info : Peak(s) manually integrated

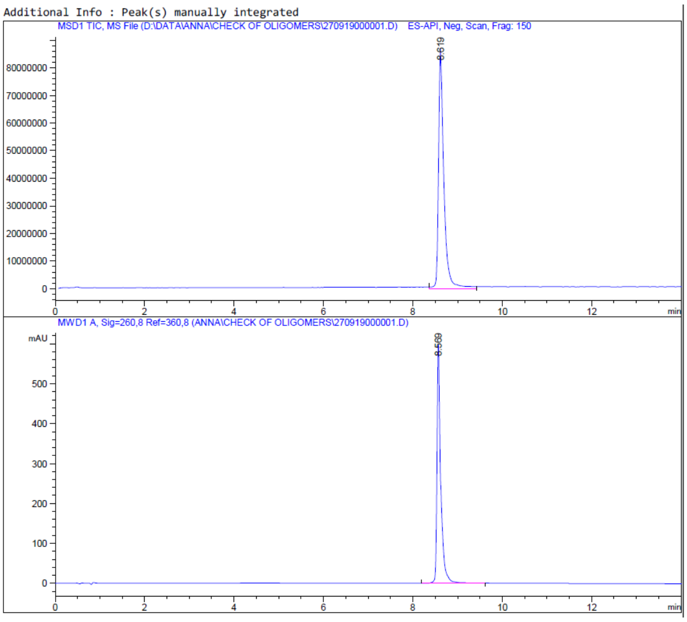

Signal 1: MSD1 TIC, MS File

Peak #	RetTime [min]	Type	Width [min]	Area	Height	Area %
1	8.619	VV	0.1201	7.26881e8	8.68373e7	100.0000

Totals : 7.26881e8 8.68373e7

Signal 2: MWD1 A, Sig=260,8 Ref=360,8

Peak #	RetTime [min]	Type	Width [min]	Area [mAU*s]	Height [mAU]	Area %
1	8.569	BB	0.0851	3551.79785	599.69592	100.0000

Totals : 3551.79785 599.69592

*** End of Report ***

Deconvolution of Spectrum # 1 @ 8.571 - 8.695 min

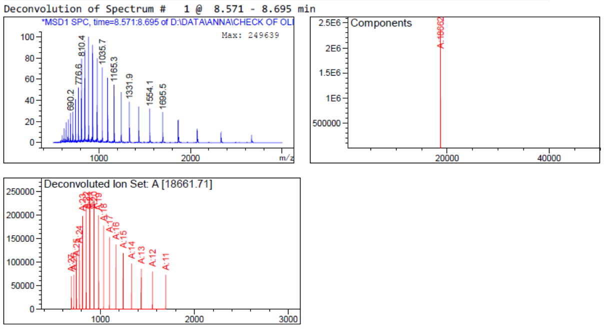

Component	Molecular Weight	Absolute Abundance	Relative Abundance
A	18661.71	2344840	100.00

*** End of Report ***

LC-MS chromatogram of hp-132-1

Additional Info : Peak(s) manually integrated

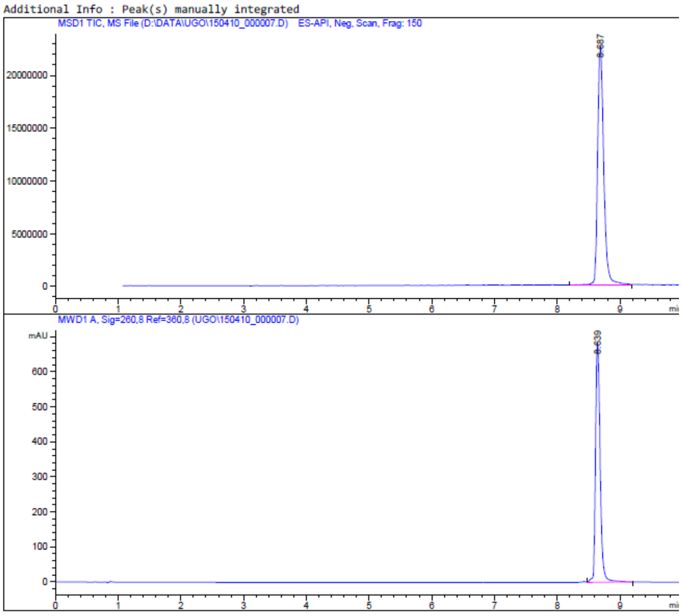

Signal 1: MSD1 TIC, MS File

Peak #	RetTime [min]	Type	Width [min]	Area	Height	Area %
1	8.687	BV	0.1086	1.55827e8	2.31096e7	100.0000

Totals : 1.55827e8 2.31096e7

Signal 2: MWD1 A, Sig=260,8 Ref=360,8

Peak #	RetTime [min]	Type	Width [min]	Area [mAU*s]	Height [mAU]	Area %
1	8.639	VB	0.0793	3481.52246	684.46436	100.0000

Totals : 3481.52246 684.46436

Deconvolution of Spectrum # 1 @ 8.646 - 8.745 min

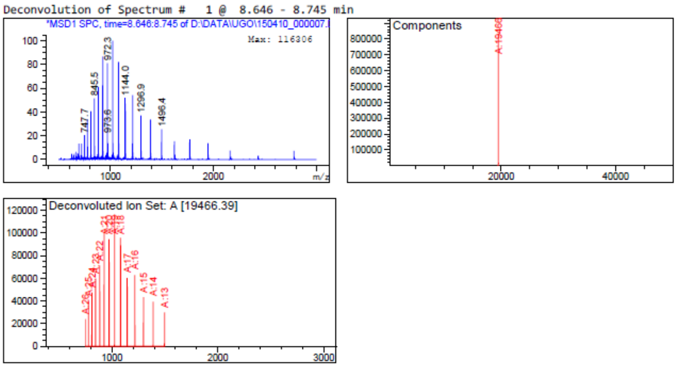

Component	Molecular Weight	Absolute Abundance	Relative Abundance
A	19466.39	832587	100.00

*** End of Report ***

LC-MS chromatogram of hp-132-2

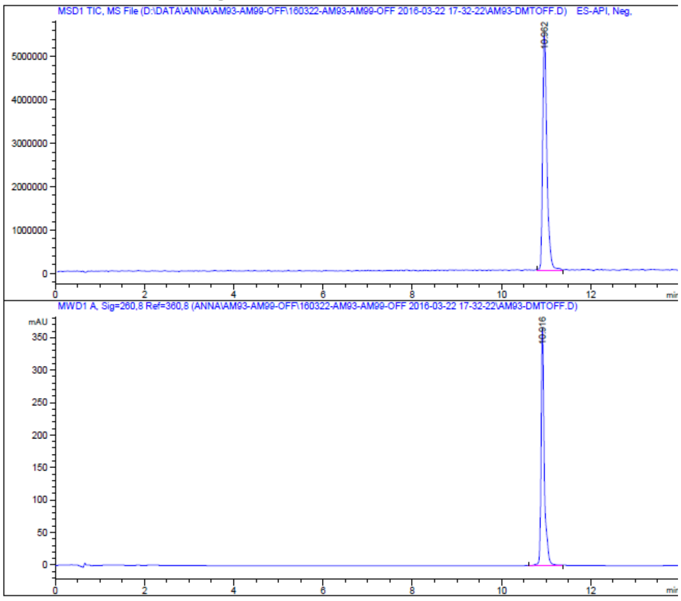

Signal 1: MSD1 TIC, MS File

Peak #	RetTime [min]	Type	Width [min]	Area	Height	Area %
1	10.962	BV	0.0974	3.62951e7	5.64529e6	100.0000
Totals :				3.62951e7	5.64529e6	

Signal 2: MWD1 A, Sig=260,8 Ref=360,8

Peak #	RetTime [min]	Type	Width [min]	Area [mAU*s]	Height [mAU]	Area %
1	10.916	VB	0.0747	1845.57593	367.09244	100.0000
Totals :				1845.57593	367.09244	

Deconvolution of Spectrum # 1 @ 10.902 - 11.002 min

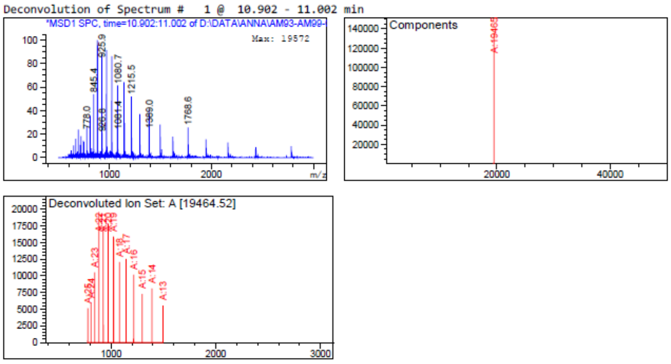

Component	Molecular Weight	Absolute Abundance	Relative Abundance
A	19464.52	135454	100.00

*** End of Report ***

Supplementary Figure S1: Chromatograms of miR-CLIP probes and wild type controls

MiRCLIP probes (see **Fig. 1b**) and unmodified pre-miRNAs were prepared and purified as described in Methods. LC-MS chromatograms shown above confirm purity and identity of the RNAs.

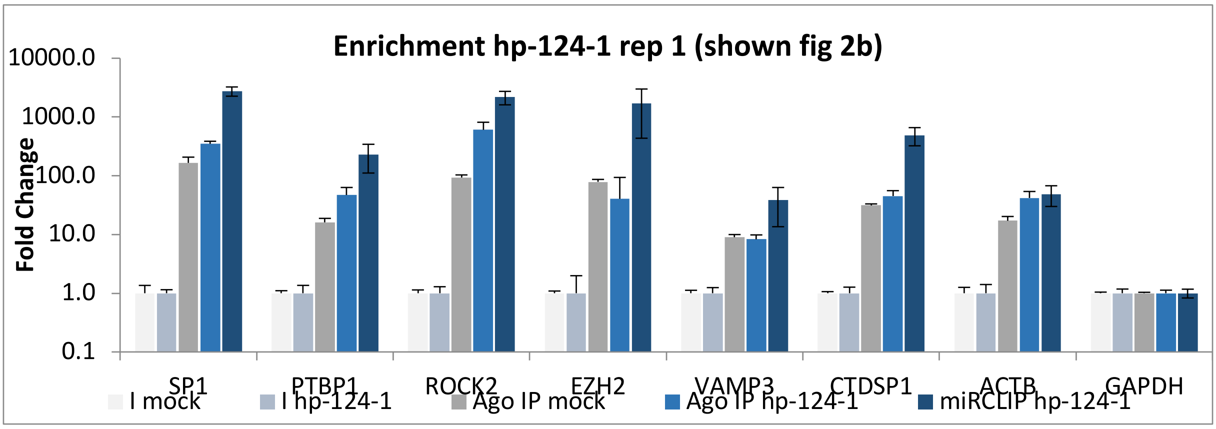

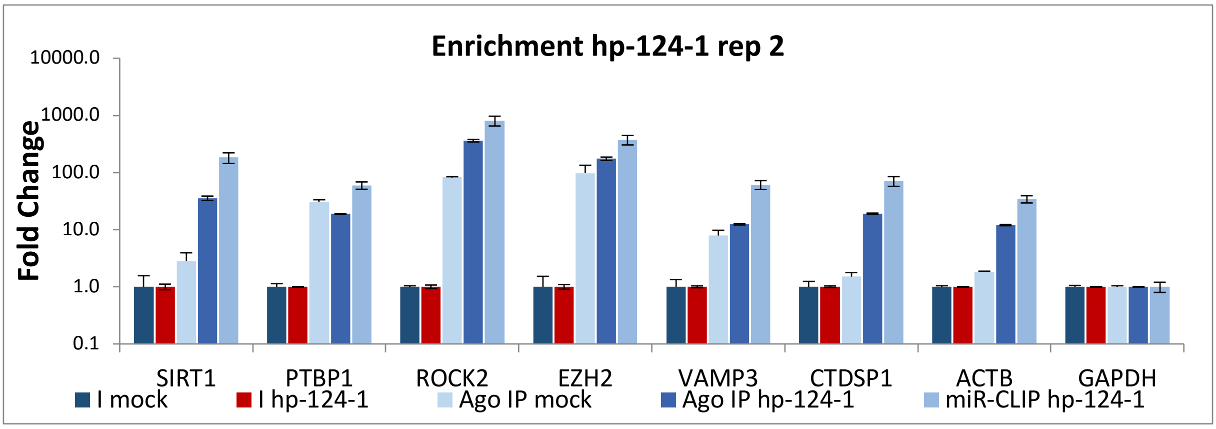

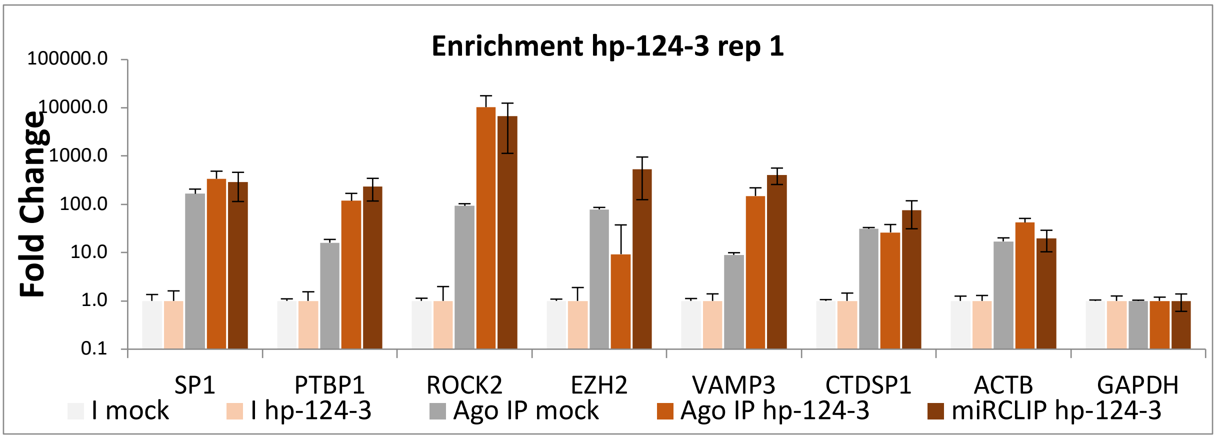

Supplementary Figure S2: miR-CLIP enrichments of all the miR-CLIP replicates sequenced.

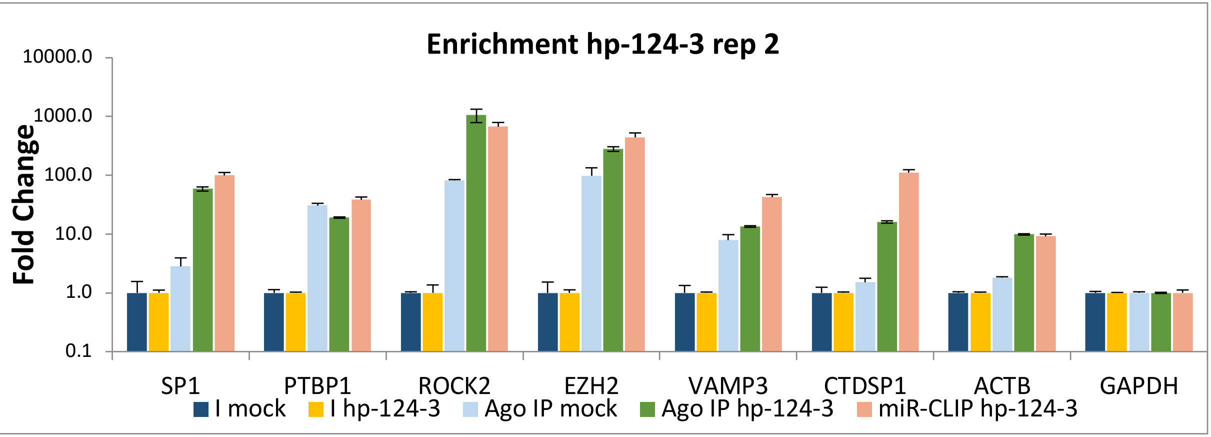

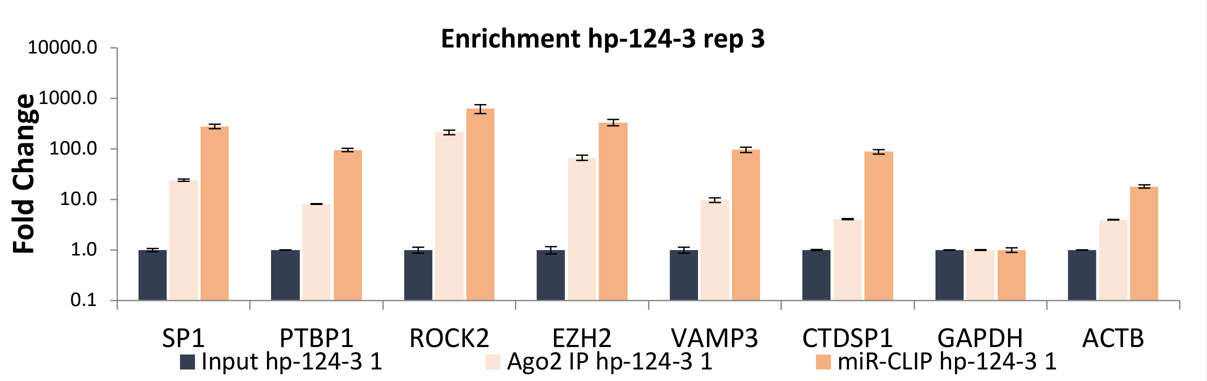

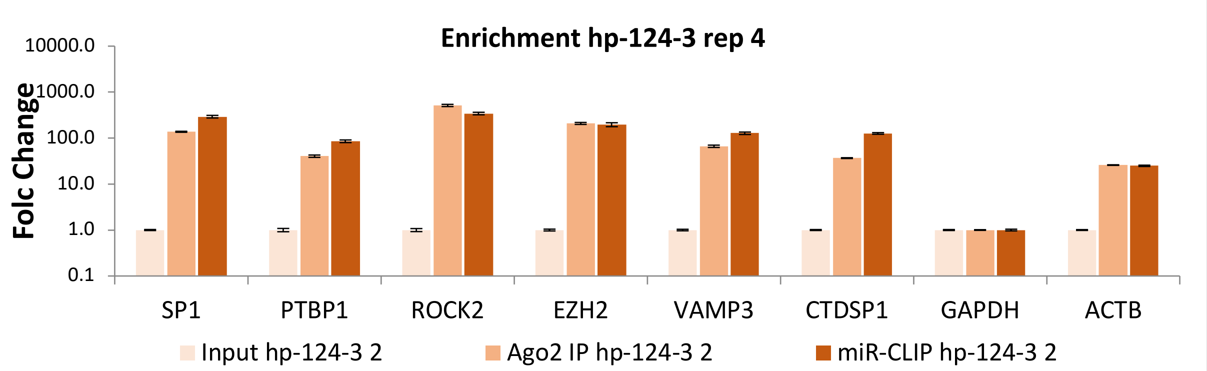

Supplementary Figure S2 (continued): miR-CLIP enrichments of all the miR-CLIP replicates sequenced.

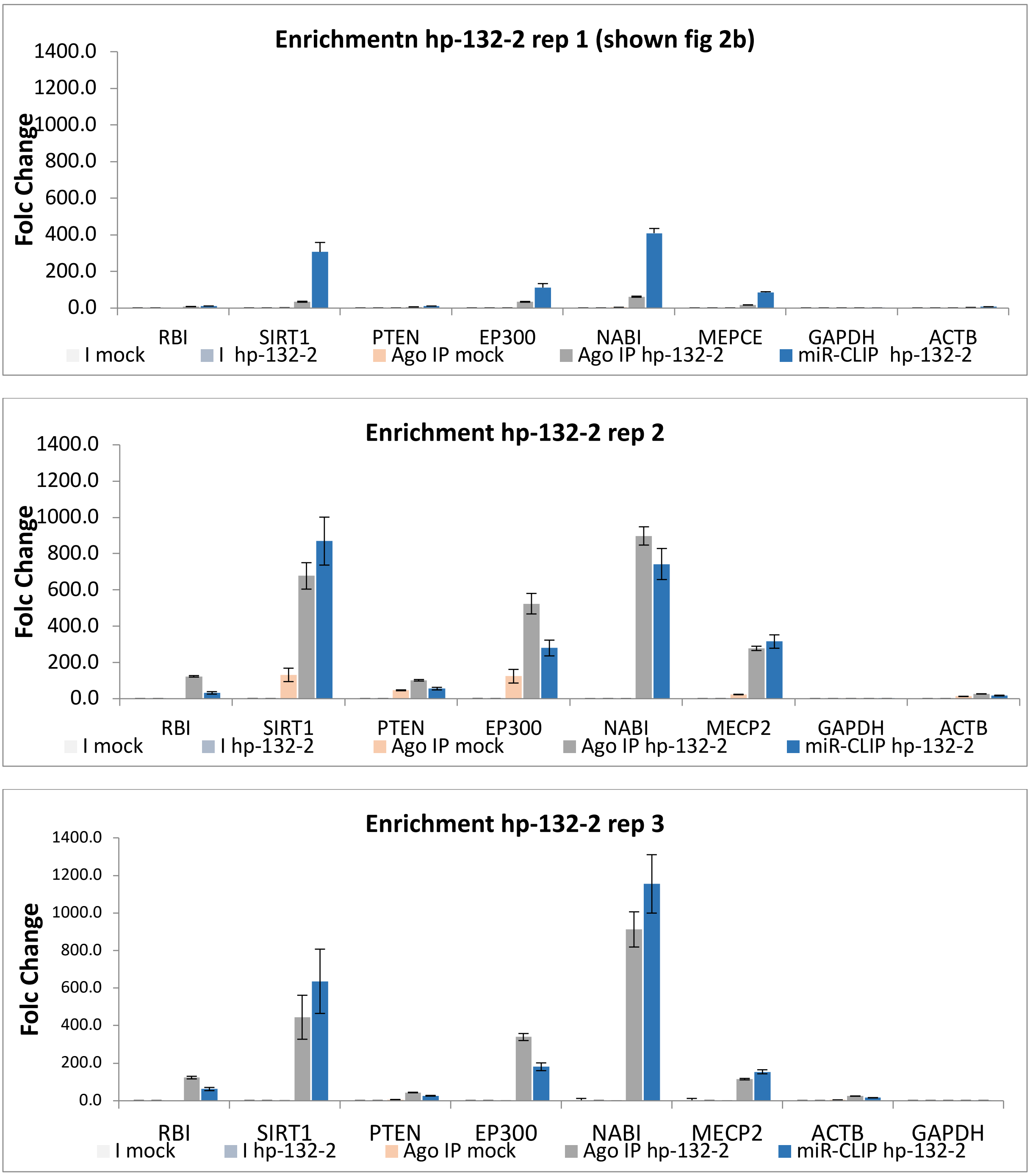

Supplementary Figure S2: miR-CLIP enrichments of all the miR-CLIP replicates sequenced.

Individual experimental replicates of enrichment experiments for six control targets for hp-miR-124-1, hp-miR-124-3 and hp-miR-132-2 (see **Figs. 2b**, **2c**); "I" is Input. Error bars show standard deviation of 3 technical replicates.

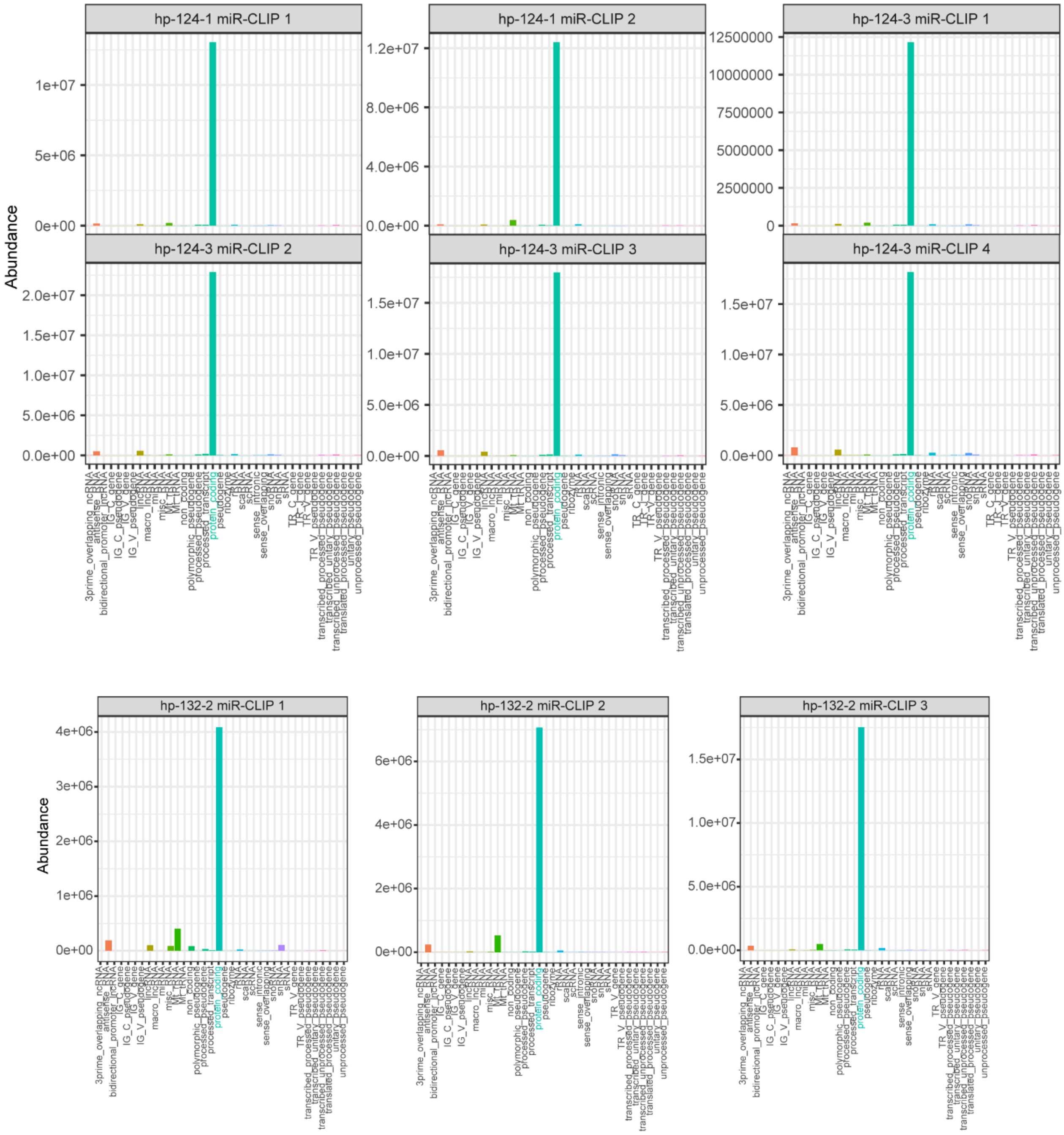

Supplementary Figure S3: Classes of RNAs identified by RNA sequencing from miR-CLIP experiments.

Hp-124-1, hp-124-3 and hp-132-2 miR-CLIP probes mainly captured protein coding RNAs from HEK293T cells. Plots show transcript counts of various RNA classes estimated by Salmon(1).

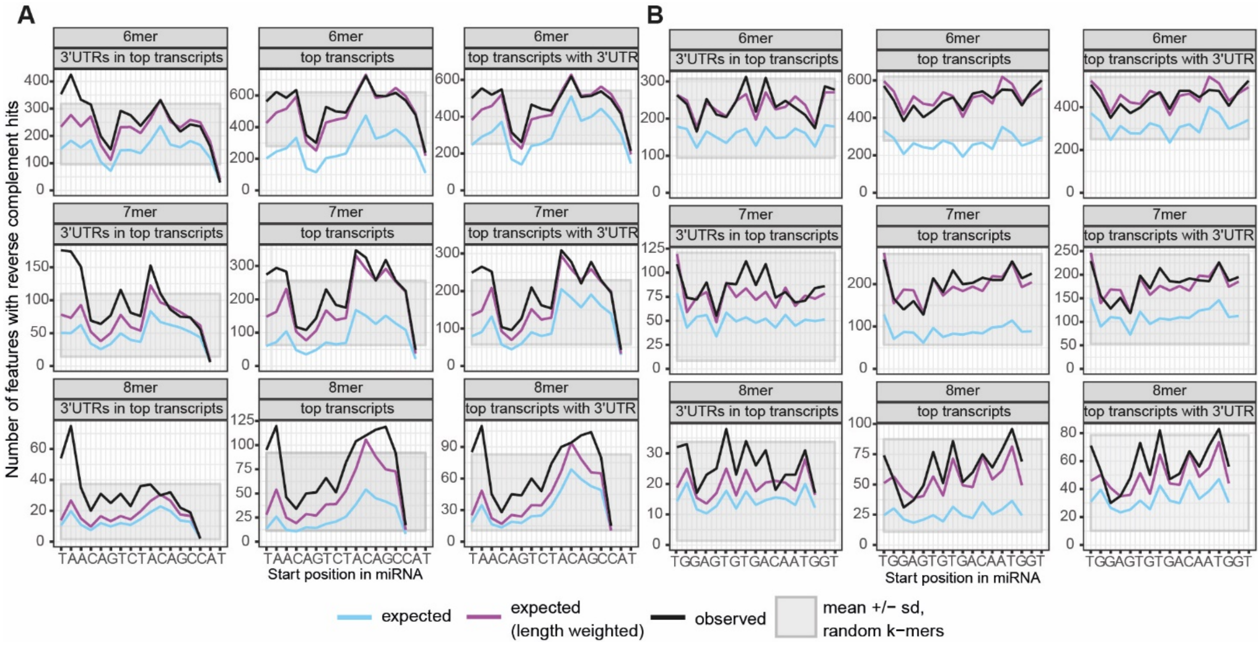

Supplementary Figure S4: Reverse complementarity of the miR-CLIP-captured targets to k-mers present in miR-132-3p.

Results plotted for hp-132-2 captured targets bearing reverse complementarity to miR-132-3p (A) or to miR-122-5p (B) as control. The observed number of top-ranked features with an exact reverse complement hit were compared to the number that we would expect if the hits were distributed randomly (the total number of features with hits divided by total number of features, multiplied by 1,000, “Expected"), and to the expected number if the probability of a hit was considered to be associated with the length of the transcript (“Expected, length weighted”). In the latter case, we fit a logistic regression model of the probability of having at least one exact hit as a function of the feature length, and then used this model to predict the probability of each feature having an exact hit. The grey box represents the mean +/- one standard deviation of the number of hits observed across 250 randomly selected k-mers.

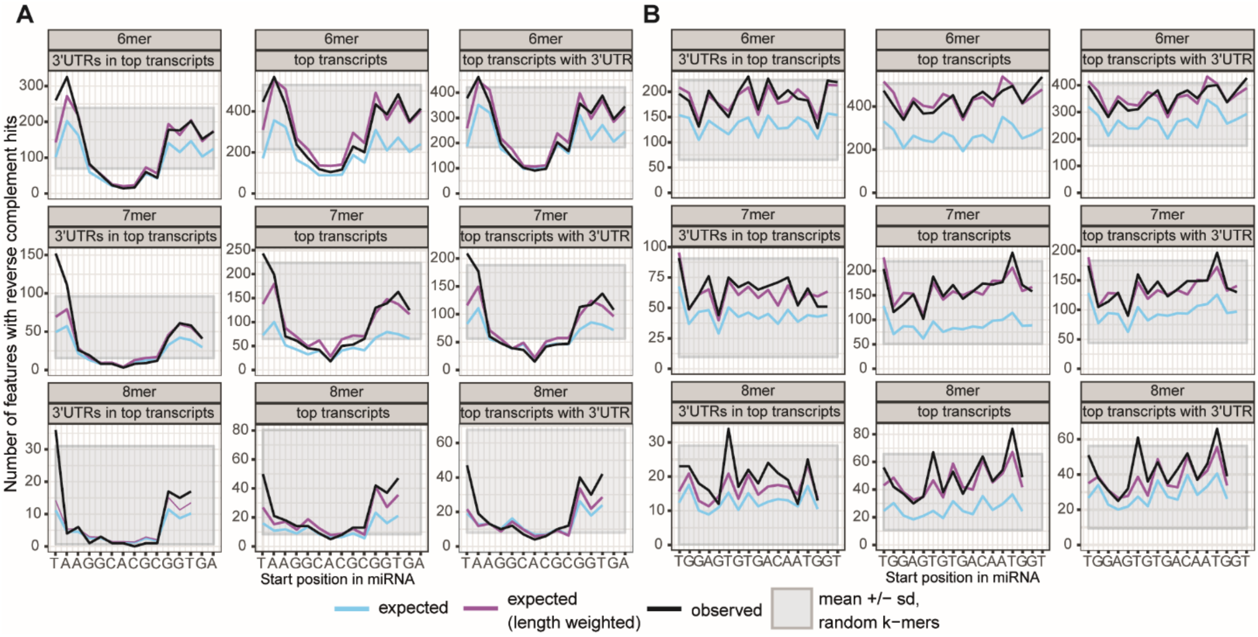

Supplementary Figure S5: Reverse complementarity of the miR-CLIP-captured targets to k-mers from miR-124-3p.

Results plotted for hp-124-3 captured targets bearing reverse complementarity to miR-124-3p (A) or to miR-122-5p (B) as control. The observed number of top-ranked features with an exact reverse complement hit were compared to the number that we would expect if the hits were distributed randomly (the total number of features with hits divided by total number of features, multiplied by 1,000, “Expected"), and to the expected number if the probability of a hit was considered to be associated with the length of the transcript (“Expected, length weighted”). In the latter case, we fit a logistic regression model of the probability of having at least one exact hit as a function of the feature length, and then used this model to predict the probability of each feature having an exact hit. The grey box represents the mean +/- one standard deviation of the number of hits observed across 250 randomly selected k-mers.

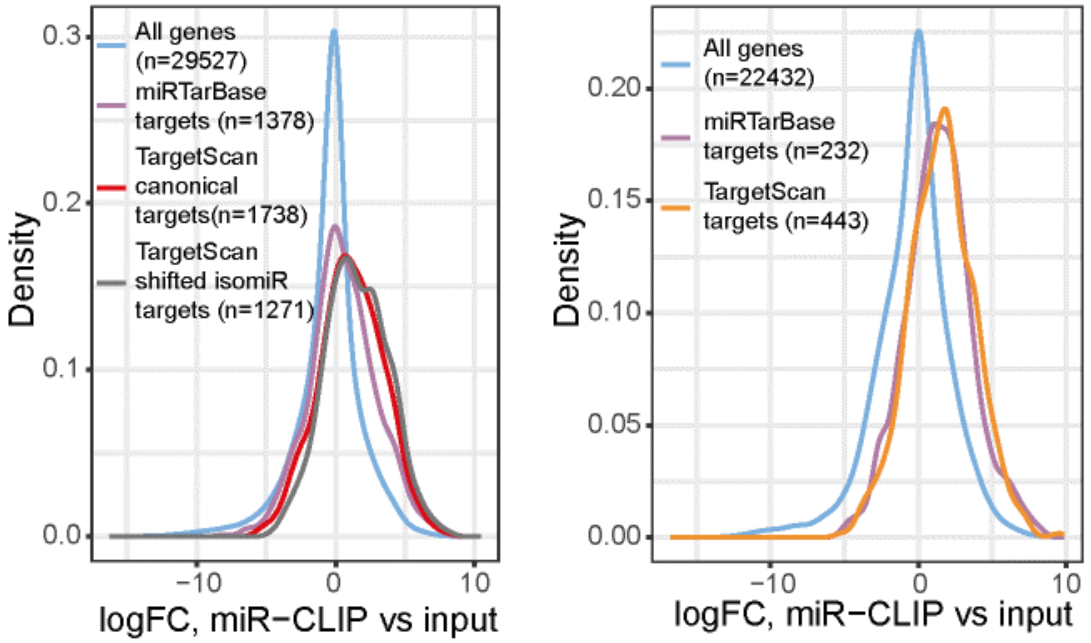

	WilcoxP
miRTarBase	1.943E-48
TS Canonical	8.475E-174
TS Shifted IsomiR	9.581E-192

	WilcoxP
miRTarBase	5.869E-27
TargetScan	2.268E-61

Supplementary Figure S6: Density plots of Targetscan-predicted and miRTarBase-reported targets from miR-CLIP samples.

Enrichment of transcripts in hp-124-3 (left) and hp-132-2 (right) miR-CLIP samples compared to the respective input samples. Blue line shows the distribution of log2 fold changes for all quantified genes. Red, grey and orange respectively show TargetScan-predicted targets. Purple lines show miRTarBase-reported targets. P-values are obtained using Wilcoxon-rank-sum-test comparing genes within the assigned subset against those outside the subset.

a) hp-124-3

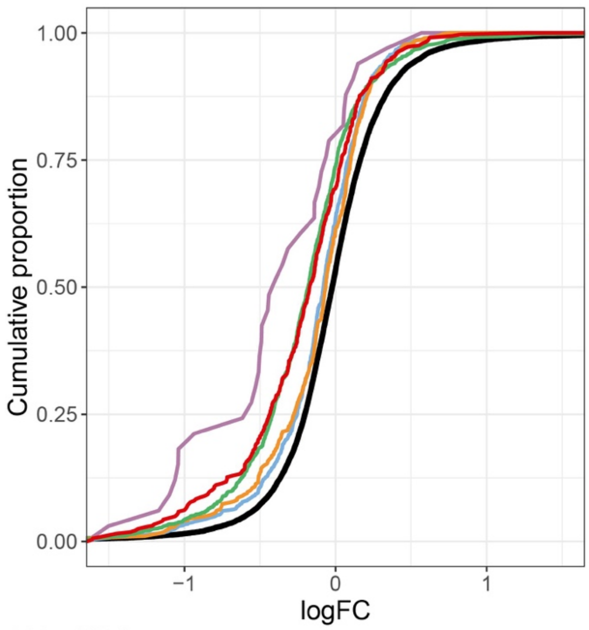

Category	Median	WilcoxP
miR-CLIP seedless in transcript	-0.07958	5.54E-11
miR-CLIP 8mer in 3'UTR	-0.39823	3.53E-06
miR-CLIP seedless in 3'UTR	-0.06561	4.11E-05
miRTarBase	-0.18096	2.51E-82
Targetscan 8mer	-0.17092	1.40E-18

b) hp-132-2

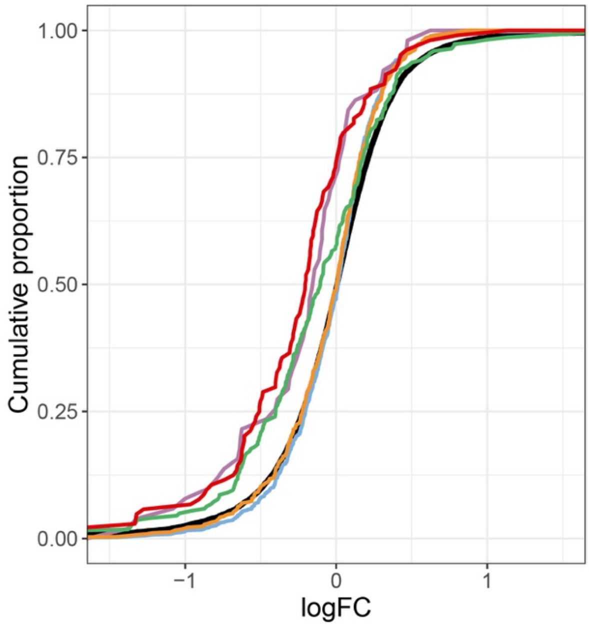

Category	Median	WilcoxP
miR-CLIP seedless in transcript	0.01168	2.79E-01
miR-CLIP 8mer in 3'UTR	-0.15225	6.59E-04
miR-CLIP seedless in 3'UTR	0.00173	1.02E-01
miRTarBase	-0.10509	1.75E-04
TargetScan 8mer	-0.20344	1.07E-08

Supplementary Figure S7: Cumulative distributions of target fold changes after pre-miRNA treatment of HEK293T cells.

Cumulative distributions of log2 fold changes in gene abundances after 40 nM pre-miR-124 (a) and pre-miR-132 (b) transfections. The distribution of fold changes in different subsets of genes were compared to genes outside the subset: seedless target site (reverse complementarity to a 7mer or more to the miRNA's non-seed (starting from position 8 of the miRNA sequence)) in transcript or 3'UTR, miR-CLIP captured targets bearing an 8mer target site in transcript or 3'UTR predicted targets, TargetScan predicted targets. P-values are obtained by Wilcoxon rank-sum test by comparing gene in subsets to genes outside the subset.

LC-MS chromatogram of ct-GNL3L

Additional Info : Peak(s) manually integrated

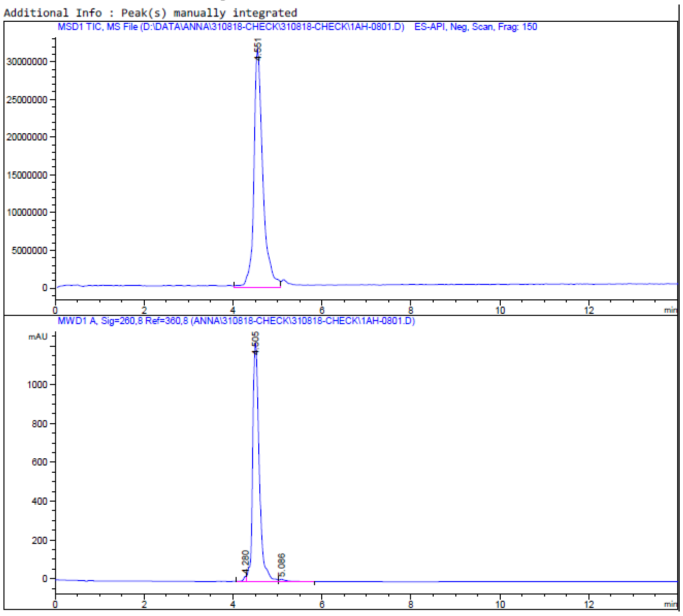

Signal 1: MSD1 TIC, MS File

Peak #	RetTime [min]	Type	Width [min]	Area	Height	Area %
1	4.551	VV	0.2093	4.44911e8	3.15524e7	100.0000

Totals : 4.44911e8 3.15524e7

Signal 2: MWD1 A, Sig=260,8 Ref=360,8

Peak #	RetTime [min]	Type	Width [min]	Area [mAU*s]	Height [mAU]	Area %
1	4.280	VV	0.0629	110.33994	26.80608	0.8622
2	4.505	VV	0.1555	1.25590e4	1226.53284	98.1361
3	5.086	VB	0.1378	128.19609	12.08385	1.0017

Totals : 1.27975e4 1265.42277

Deconvolution of Spectrum # 1 @ 4.452 - 4.651 min

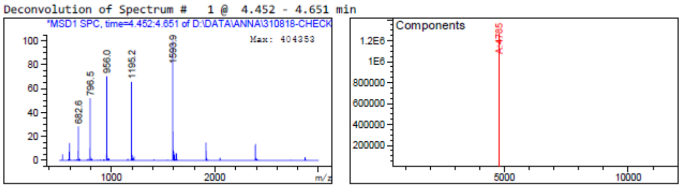

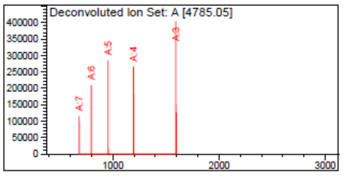

Component	Molecular Weight	Absolute Abundance	Relative Abundance
A	4785.05	1265686	100.00

*** End of Report ***

Supplementary Figure S8: LC-MS chromatograms of reagents used for the *in vitro* cross-linking experiment.

LC-MS chromatogram gs-132-2

Additional Info : Peak(s) manually integrated

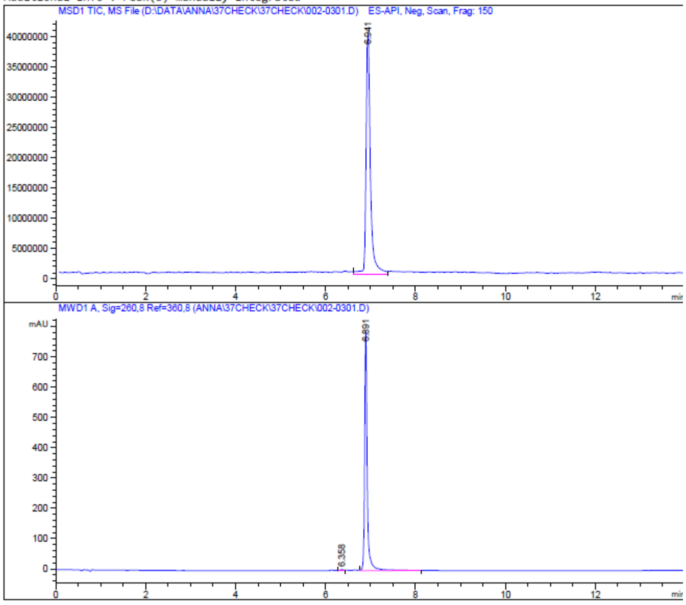

Signal 1: MSD1 TIC, MS File

Peak #	RetTime [min]	Type	Width [min]	Area	Height	Area %
1	6.941	VV	0.0921	2.68355e8	4.05827e7	100.0000

Totals : 2.68355e8 4.05827e7

Signal 2: MWD1 A, Sig=260,8 Ref=360,8

Peak #	RetTime [min]	Type	Width [min]	Area [mAU*s]	Height [mAU]	Area %
1	6.358	VV	0.0700	20.62664	4.29478	0.6269
2	6.891	VB	0.0621	3269.49512	792.69806	99.3731

Totals : 3290.12176 796.99283

Deconvolution of Spectrum # 1 @ 6.900 - 7.000 min

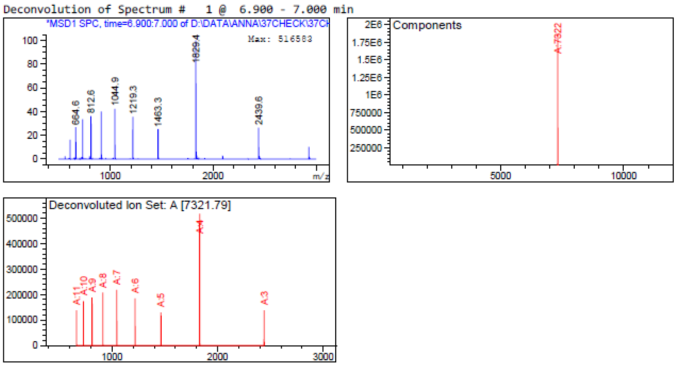

Component	Molecular Weight	Absolute Abundance	Relative Abundance
A	7321.79	1876064	100.00

*** End of Report ***

Supplementary Figure S8 (continued): LC-MS chromatograms of reagents used for the *in vitro* cross-linking experiment

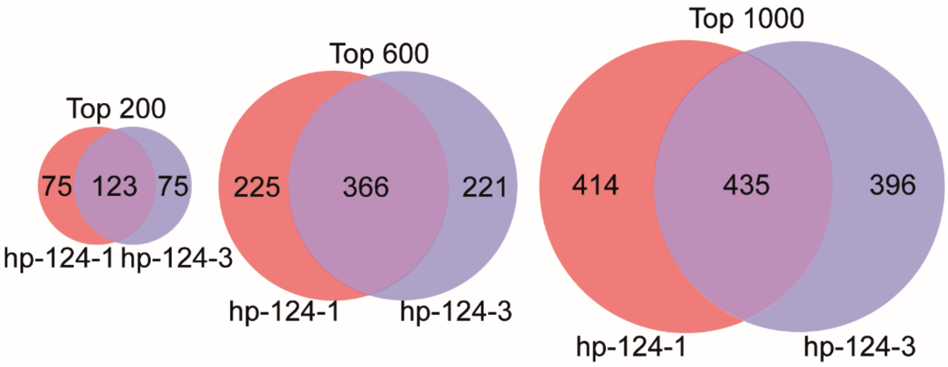

Supplementary Figure S9: Overlap of datasets containing RNAs captured by hp-124-1 and hp-124-3.

Three overlapping datasets of RNAs (see Fig. S3) captured by hp-124-1 and hp-124-3 miR-CLIP probes: top 200 (left), 600 (middle) or 1000 (right) miR-CLIP/input enriched transcripts. Multiple transcripts mapping to the same genomic locus were counted once in assigning miRNA targets.

Supplementary Figure S10: Small RNA-Seq read alignments to miR-124 (separate file)

A spreadsheet file depicting all unique read alignments to the miR-124-3p sequence, including two nucleotides on either side (present in all three genomic loci coding for the miRNA). Hyphens within read sequences denote deletions and dots are used on either end to fill up and align shorter sequences with the 26-nt reference sequence at the top. Numbers of reads supporting each alignment are indicated. Tabs "Replicate 1" to "Replicate 3" report the alignments and numbers individually for each alignment, whereas tab "Sum" reports aggregated counts on all alignments observed in either replicate. Tab "Summary" lists summary data such as sums, means and standard deviations for the main miR-124 and iso-miR-124 species.

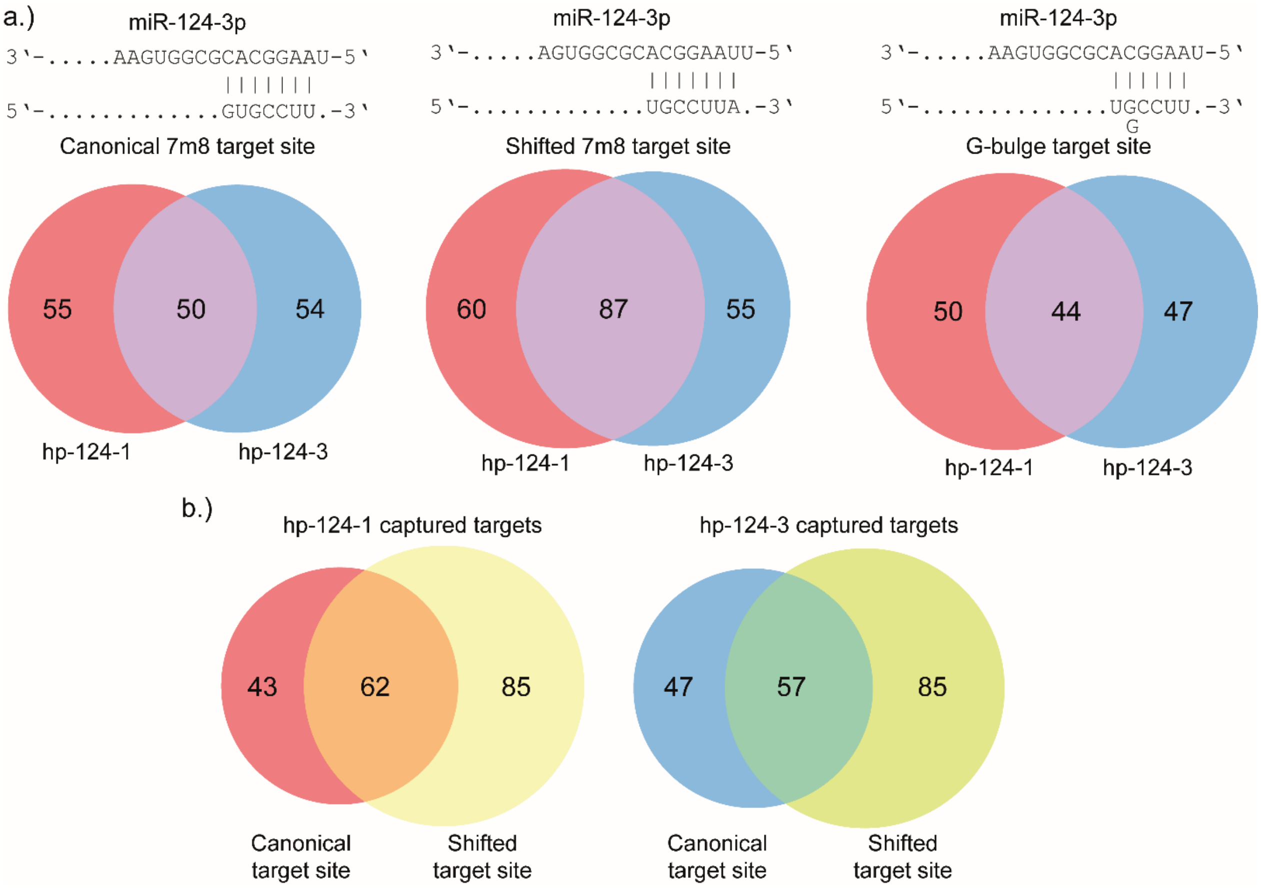

Supplementary Figure S11: Overlapping datasets of target RNAs bearing selected seed target motifs, captured by the miR-124 miR-CLIP probes hp-124-1 and 124-3.

a) Upper panel: sequence alignments for selected seed target motifs of miR-124 and *iso*-miR-124. Lower panel: overlapping datasets of target transcripts bearing the selected seed target motifs of miR-124 and *iso*-miR-124, captured by hp-124-1 or hp-124-3 (top 1000 enriched miR-CLIP/input). b) Overlap of datasets containing target transcripts bearing canonical or shifted seed target motif, captured by the indicated miR-CLIP probe (top 1000 enriched miR-CLIP/input). Multiple transcripts mapping to the same genomic locus were counted once in assigning miRNA targets.

<table>
<tr><th>Target mRNA</th><th>Predicted, conserved target sites</th></tr>
<tr><td>Position 178-185 of APEX2 3'UTR
• miR-124: 8mer site
• iso-miR-124: 8mer site</td><td><pre>5'-GGUGAGCUUCUUGUGCCUUAAU
    ::   :   :   :::::::::
3'-CCGUAAGUGGCGCACGGAAU   (miR-124)
3'-CCGUAAGUGGCGCACGGAAUU (iso-miR-124)</pre></td></tr>
<tr><td>Position 131-137 of LMNB1 3'UTR
• iso-miR-124: 7merA1 site
• miR-124: 2-6 nt binding</td><td><pre> 5'-UGCAAAUCUGAUGGCCUUAA
              : :   :::::::
3'-CCGUAAGUGGCGCACGGAAUU (iso-miR-124)
3'-CCGUAAGUGGCGCACGGAAU   (miR-124)</pre></td></tr>
</table>

Supplementary Figure S12: Sequence alignments for predicted canonical targeting of LMNB1 and APEX2 by miR-124 and iso-miR-124.

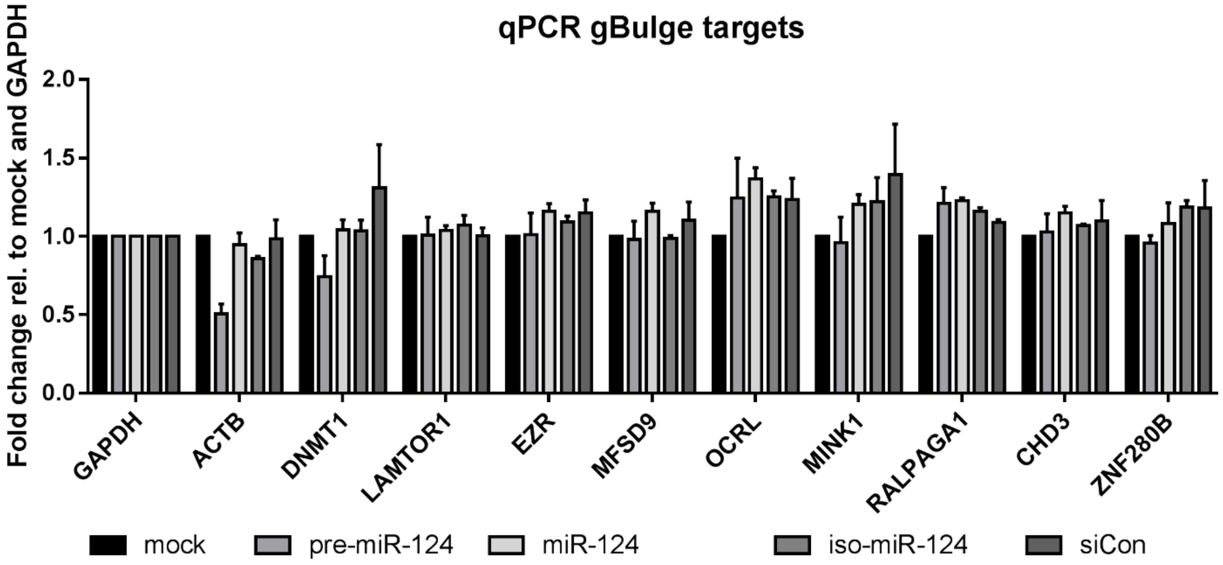

Supplementary Figure S13: Transcript level of G-bulge targets upon miR-124 transfection determined by SYBR green RT-qPCR.

Error bars show standard deviation of 3 biological replicates. The transcript levels of the G-bulge targets after treating cells with miR-124 duplexes did not significantly differ from transcript levels after siCon neg. control treatment (significance between siCon and miR-124 or *iso*-miR-124 respectively calculated by 2-way ANOVA Dunnett test).

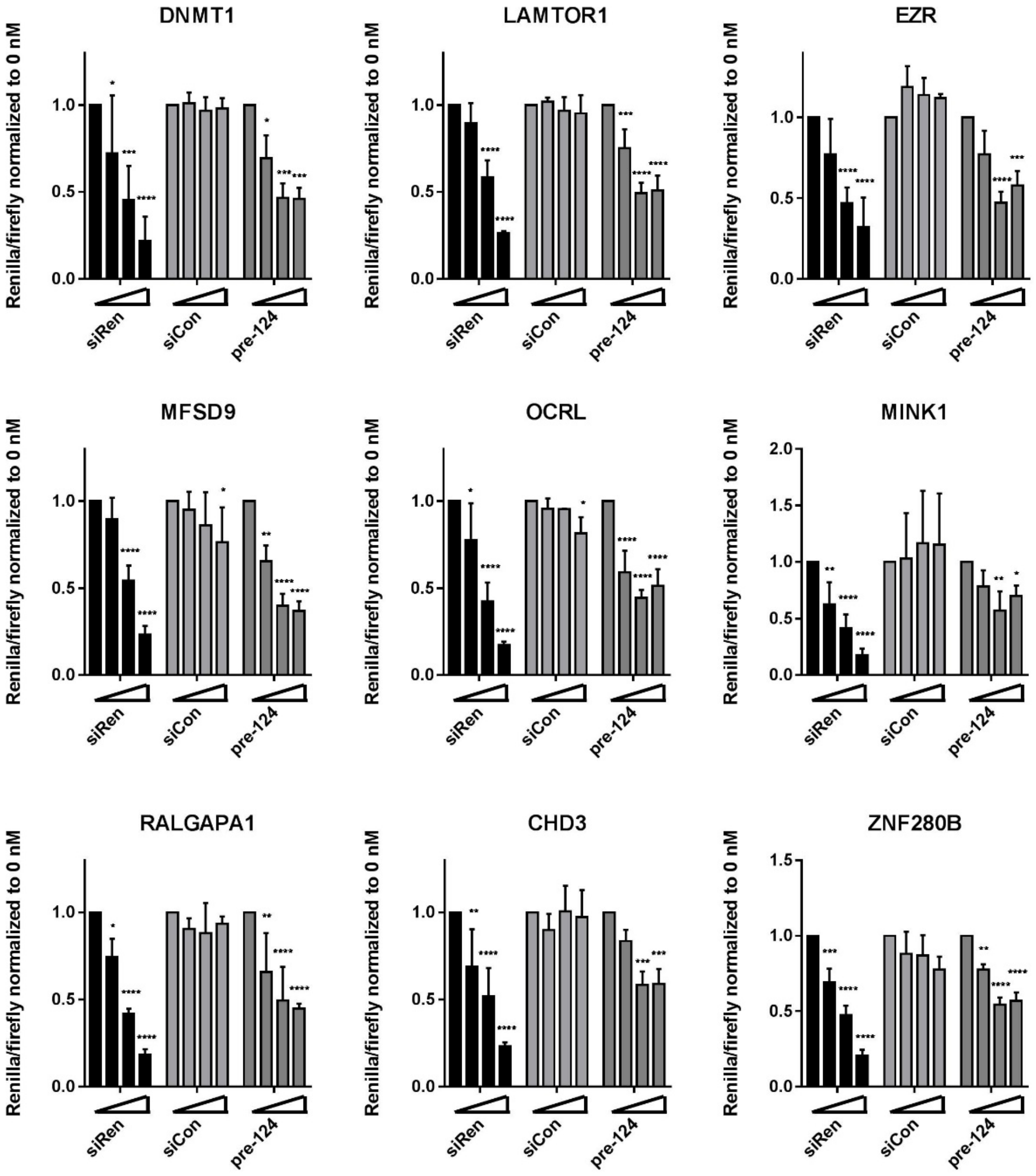

Supplementary Figure S14: Luciferase reporter assays for effect of pre-mir-124 on 9 discovered G-bulge target sites.

Reporters carrying an indicated transcript were co-transfected, with 0 nM, 2.5 nM, 10 nM or 40 nM, of acting as a positive control anti-Renilla siRNA (siRen), acting as negative control randomized RNA duplex (siCon) or hairpin of pre-mir-124 (pre-124). Luciferase activity represents Renilla/firefly values normalized to 0 nM and is expressed as mean ± S.D. N=3. Asterisks indicate significance of each treatment compared to respective 0 nM dose calculated by 2-way ANOVA Dunnett test whereas * = P < 0.05, ** = P ⩽ 0.01, *** = P ⩽ 0.001, **** = P ⩽ 0.0001, not significant not indicated.

G-Bulge target: miR-124	G-Bulge target: *iso*-miR-124
...NNNNNNNNNNNNNUG^{G}CCUUGNN<br>:: ::::<br>3'-CCGUAAGUGGCGCAC GGAAU (miR-124)<br>76 54321	...NNNNNNNNNNNNNUGGCCUUGNN<br>:::::<br>3'-CCGUAAGUGGCGCACGGAAUU (*iso*-miR-124)<br>7654321

Supplementary Figure S15: Alignment of proposed binding of miR-124 and *iso*-miR-124 to DNMT1.

DNMT1 site in a luciferase reporter is inhibited by miR-124 but not by *iso*-miR-124 in HEK293T cells. Left: presumed targeting of G-bulged site in the *DNMT1* 3'UTR by miR-124. Right: the same site produces a G-U wobble at g2 (also in *EZR*, *MFSD9*, *MINK1*, *ZNF28*).

Supplementary Tables

Supplementary Table S1: Characterization of synthesized oligoribonucleotides

	ORN	Sequence (5' to 3')	Length	Mass calc.	Mass found
1	pre-miR-124	CGUGUUCACAGCGGACCUUGAUUUAAAUGUCCAUACAA-UUAAGGCACGCGGUGAAUGCC-3'	59	18903.40	18901.51
2	hp-124-1	CGUGUUCACAGCGGACCUUGAUUUAAAUGUCCAUACAA-UUAAGGXACGCGGUGYAUGCC-3'	59	19707.37	19706.10
3	hp-124-3	CGUGUUCACAGCGGACCUUGAUUUAAAUGUCCAUACAA-UUAYGGCACGXGGUGAAUGCC-3'	59	19707.37	19707.39
4	pre-miR-132	ACCGUGGCUUUCGAUUGUUACUGUGGGAACUGGAGGU-AACAGUCUACAGCCAUGGUCG-3'	58	18663.20	18661.71
5	hp-132-1	ACCGUGGCUUUCGAUUGUUACUGUGGGAACUGGAGGU-AYCAGUCUACAGXCAUGGUCG-3'	58	19467.17	19466.39
6	hp-132-2	ACCGUGGCUUUCGAUUGUUACUGUGGGAACUGGAGGU-AACYGUCUACAGCXAUGGUCG-3'	58	19467.17	19464.52
7	ct-GNL3L	UGGCUGUGGACUGUU-3'	15	4785.90	4785.05
8	gs-132-2	UAACYGUCUACAGCCAUGGUCG-3'	22	7322.65	7321.79
X=C-biotin, Y=A-psoralen (trioxsalen)					

Supplementary Table S2: Luciferase plasmid reporter inserts

Plasmid name	*Reporter type*	*Insert sequence*	*5' Restriction site*	*3' Restriction site*	*Insert Fw primer binding site*	*Insert Rv primer binding site*
1x sensor for miR-124-3p	psiCHECK2	ggcctgcaggtgggcgccccgaccacgacttggcttccttGGCATTCACCGCGTGCCTTAaaaaacctgccttcctgcagccacacacccacccggggtg	XhoI: CTCGAG	NotI: GCGGCCGC	CCTCCACTTCAGCCAGGA	TGAGTCTTCGGACCTCGC
1x sensor for miR-132-3p	psiCHECK2	acatagtcttttatgtaatttactggcatatgttttgtaCGACCATGGCTGTAGACTGTTAaatgactggatatcttccttcaacttttgaaatacaaaa	XhoI: CTCGAG	NotI: GCGGCCGC	CCTCCACTTCAGCCAGGA	TGAGTCTTCGGACCTCGC
GNL3L miR-132 binding site	psiCHECK2	TTACATCCTAAGAGTGGAACCATGTGACAAGGACTGGAGTGCCATTGGCTGTGGACTGTTCAGGCAGGGAAGTACAAGACCACTCTTGTATTCAGGGGCA	XhoI: CTCGAG	NotI: GCGGCCGC	CCTCCACTTCAGCCAGGA	TGAGTCTTCGGACCTCGC
DNMT1 G-Bulge reporter	psiCHECK2	TTTTAACATGTCAATCTGTCCGTTCACATGTGTGGTACATGGTGTTTGTGGCCTTGGCTGACATGAAGCTGTTGTGTGAGGTTCGCTTATCAACTAATGATTT	XhoI: CTCGAG	NotI: GCGGCCGC	CCTCCACTTCAGCCAGGA	TGAGTCTTCGGACCTCGC
LAMTOR1 G Bulge reporter	psiCHECK2	AGAGGAAGGCTAGAAGCCTGAGCAAGTGAGGGTAGAACCTTTTGGGACTGGCCTTTGAAGCTCTGGCCAGGGATGGGGTGGGGGCCAAAAGGACAGAGCCTGG	XhoI: CTCGAG	NotI: GCGGCCGC	CCTCCACTTCAGCCAGGA	TGAGTCTTCGGACCTCGC
EZR G Bulge reporter	psiCHECK2	AGAGACTCTGTTTGCTTGTGTTTGTTTGTTTGCAGTCTCTCTCTGCCATGGCCTTGGCAGGCTGCTGGAAGGCAGCTTGTGGAGGCCGTTGGTTCCGCCCACT	XhoI: CTCGAG	NotI: GCGGCCGC	CCTCCACTTCAGCCAGGA	TGAGTCTTCGGACCTCGC
MFSD9 G Bulge reporter	psiCHECK2	AAATGTTAAGTGCTTTCGAGGAGACCAAACTATTATTAATATATAAAATGGCCTTGCCCTTAAGGAGCAAATTAAATCTCATGGAGATTAGACTCAAAAGGCA	XhoI: CTCGAG	NotI: GCGGCCGC	CCTCCACTTCAGCCAGGA	TGAGTCTTCGGACCTCGC
OCRL G Bulge reporter	psiCHECK2	GCATTTGTGCATTTCTACTACAATGGCATCTTTATGTCTCTGTAACATTGGCCTTTTCATGGCTCCACACTGGGTGGAACCATATTCTCTTAGATCACATTTA	XhoI: CTCGAG	NotI: GCGGCCGC	CCTCCACTTCAGCCAGGA	TGAGTCTTCGGACCTCGC
MINK1 G Bulge reporter	psiCHECK2	ATATAGTGTGAGCAGCAAGTAACCCTTCTCCCTCCCCCCCCACCCCTCCTCAATGTAGTGGCCTTGGATATCCTGTTTGTTAATAAAGACAATTCAACCAGCT	XhoI: CTCGAG	NotI: GCGGCCGC	CCTCCACTTCAGCCAGGA	TGAGTCTTCGGACCTCGC
RALGAPA1 G Bulge reporter	psiCHECK2	TTGCATTTCAAGTGGTATCACTGTTAAATGCCATTTGTTTTCAGATTGTGGCCTTTTATTATTGGCTGCTAGATCCTGGTGTTTCTATGTTCTTTTTTAAGCA	XhoI: CTCGAG	NotI: GCGGCCGC	CCTCCACTTCAGCCAGGA	TGAGTCTTCGGACCTCGC
CHD3 G Bulge reporter	psiCHECK2	CTGCAGAATCAGCTCTGTCTCATGTGGAAGTGGAGAATCAGCCTTGCCTGGCCTTTAGGAACTTTTGTGGGGAAGAGAGCTTTGAAGAGAGGAGGGGGACTTT	XhoI: CTCGAG	NotI: GCGGCCGC	CCTCCACTTCAGCCAGGA	TGAGTCTTCGGACCTCGC
ZNF280B G Bulge reporter	psiCHECK2	GTCATGCTTCTCCACTGTTTTCCCCAATGATGCACTATTTTGACACACTGGCCTTGCTATTCCTTAAGCAAATCACATTCTTCCCAATCCAAAGACTTTGCAC	XhoI: CTCGAG	NotI: GCGGCCGC	CCTCCACTTCAGCCAGGA	TGAGTCTTCGGACCTCGC

Supplementary Table S3: qPCR primers used for SYBR green qPCR

	FORWARD	REVERSE
**ACTB**	CCAACCGCGAGAAGATGA	CCAGAGGCGTACAGGGATAG
**APEX2**	AGCTGGATGCGGATATCGT	GGGGCTCTGTCAGTGCAT
**ARHGEF26**	CAACTTCTAGCCCCAAAACG	AGAAAAATCCACCTCGCTCTC
**ATP6V0E1**	CTCACTGTGCCTCTCATTGTG	CACCAACATGGTAATGATAACTCC
**CBY1**	CGGCTGAAAGTGGACATCTTA	TTCCTTCTCCATTAAGTGGGATT
**CD164**	CTTCCAAGACAGTTACTACATCAGGT	CCAGGACAATTCCTCCAATG
**CERS2**	TCTGATGTCAAGCGAAAGGA	GAGTCCCAGCTCGGATGTAA
**CPNE8**	TTCCAGCTCTAGGATTTGGTG	GGGTTTTGAGGATTCCCATT
**CREB3L2**	CTACACAGCCTCCGTGGTG	CTCTGGGGGAGAATGTTCCT
**CRY2**	CTCATACCCTCTATGACCTGGAC	CTGAAAGCGCTTGTATGTAAGG
**CTDSP1**	TCGGCCGTCATTACTCAGAT	CTGCTGACTTCTGGTCACCTT
**CTDSPL**	CGGTTGAAATCGATGGAACT	GGCAGTAAAGAGCACACATTCA
**EP300**	GATCTGTGTCCTTCACCATGAG	AAACAGCCATCACAGACGAA
**EZH2**	CGAAGACTTCAGCAGACACAA	TGTCCACGTTAACTCGCATT
**FRS2**	GGAAGAATCTACACATCCTTTGC	TCTTGCACACCTGTAGTGTTGA
**GAPDH**	AGCCACATCGCTCAGACAC	GCCCAATACGACCAAATCC
**GNL3L**	GGAGGAGTTTGAGCATAAGGAG	TCCTTGTAATAAGCCTTCCTCGT
**ITGB1**	TTCTCCAGAAGGTGGTTTCG	ACACCAGCAGCCGTGTAAC
**LAMC1**	CTGTTACTAGCCTCCTCAGCATTA	GCTTATTCAGGTCCACTGTATCC
**LMNB1**	TGCCCAAGTGAGGCTGTATAA	TGACAGTCTGGCATTCTCAAG
**LRCH1**	GCAGTTTACAATCCGGAGGA	CCTTCAATCTCATCTCAATGCTC
**MDC1**	GGCTGCGGTTGTGAAAGA	CTGGGTGTCCTCCATGATCT
**MECP2**	TGCTTGCCCTCTTTCTCTTC	GGCGAGGAGGAGAGACTG
**MYLIP**	ACAACCAGAACACTGCCAAGT	CCCCTCCAACTCCTTATGTTT
**NAB1**	AGGTTGTCTGCAGGGCTTT	AGGTTGTCTGCAGGGCTTT
**NACC2**	TCCCAAGCTCTACTCGGAAG	GTGTGATGTAGACCCCAGAGC
**POLR3G**	ACTTCGGCTGCAGAGTTTTG	AGTGGGCAAATTCTGAAAGG
**PPP1R13L**	CCCCTGTGAACGAAGGAC	GGCTGAGAGGCCTTGCTAC
**PROSER1**	CAGGTGGTTGATTTGCTGAG	GCTGGACAGCCACCATTT
**PSMA2**	GGTTGGAATGAGGGACGAC	TTTCCCATTGCTGTAGCTTTC
**PTBP1**	CGTCGTCAAAGGATTCAAGTT	TGGTTGTGCAGGTCAATGAG
**PTEN**	GCACAAGAGGCCCTAGATTTC	CGCCTCTGACTGGGAATAGT
**RAVER1**	TGACAGCTACAGCTTCGACTACC	GGGGGACATCTTGTGTCG
**RB1**	TCCTGAGGAGGACCCAGAG	AGGTTCTTCTGTTTCTTCAAACTCA
**RHOG**	CCCGCTCTCACTTCCTTCT	CTCTTCTGGACCCCTCTGG
**ROCK2**	GGAAGAAATCAGACAGCATCCTT	TGCTGAGTTCAGGTACTACAGGAG
**SIRT1**	AAATGCTGGCCTAATAGAGTGG	TGGCAAAAACAGATACTGATTACC
**SMN2**	CTGCTCCATGGAACTCTTTTCT	GAGGTGGTGGGGGAATTATC
**SNAI2**	TGGTTGCTTCAAGGACACAT	GCAAATGCTCTGTTGCAGTG
**SP1**	CTATAGCAAATGCCCCAGGT	TCCACCTGCTGTGTCATCAT
**SPG20**	GACTTTATATAGCGAAGCAAGCTACA	CAGTGCAAACTCCATCAACC
**STMN1**	GTGCTCAGAGTGTGGTCAGG	GCACAATCAACTGGGATAAGG
**SYPL1**	CGCCTTGTAAGAAGAAAGCAGTA	CATTTCCTCCCCAGAGTATCAT
**TAB2**	GCCACCAAAACCCAAAGAT	TTGGCATCTCACACTGTTCAC
**TCEAL4**	AGCGGGACATAACAACTTCG	TTCCGCCATTCCAGTGTT
**TJAP1**	ACTTGCCCCAGTCCAACC	AGTTAGCCCTCGGCTCTCC

**VAMP3**	CGAAGACTTCAGCAGACACAA	TGTCCACGTTAACTCGCATT
**ZFP62**	CTGCAGTAACTGTCTTCATGGTTT	TCCTCAGTGCTCGTCTTCAA
**ZIC2**	CCGAGAACCTCAAGATCCAC	AGCCCTCAAACTCACACTGG
**ZNF559**	CATCTTAACAGCGCGTTCC	TGAGAAAGATGATCGGGACA
**ZNF267**	GCCTTGGAATGTGAAGAGTGA	AGGTCCTTGTTATAATGCGAAAA

Supplementary Table S4: QC parameters of hp-124-1- and hp-124-3-sequenced libraries

Raw data uploaded to ArrayExpress under accession number E-MTAB-8517.

Formal name (in manuscript)	Experimental name
Hp-124-1	UP358
Hp-124-3	UP360

Sample Name	% mapped to transcript	Mapped reads	% overall Aligned	% Trimmed	% Duplicate reads	% GC	Length	Million sequences
20170719.A-UP358AgoIP_2_GATCAG_R1	43.70%	27.4	55.90%	22.30%	92.20%	51%	63 bp	62.6
20170719.A-UP358input_2_GCCAAT_R1	68.30%	27.2	81.20%	23.00%	90.40%	54%	60 bp	39.8
20170719.A-UP358miR_CLIP_2_GGCTAC_R1	39.90%	13.2	52.60%	29.00%	95.00%	47%	59 bp	33.1
20170719.A-UP360AgoIP_2_TAGCTT_R1	42.70%	19.9	57.80%	19.10%	95.10%	52%	64 bp	46.5
20170719.A-UP360input_2_CAGATC_R1	69.50%	33.7	80.60%	18.10%	93.70%	54%	63 bp	48.5
20170719.A-UP360miR_CLIP_2_CTTGTA_R1	33.10%	25	60.00%	25.80%	95.20%	48%	61 bp	75.5
20170719.A-mock_AgoIP_2_ACTTGA_R1	46.10%	23.4	61.10%	18.30%	94.00%	51%	65 bp	50.9
20170719.A-mock_input_2_ACAGTG_R1	71.70%	25.6	77.50%	15.20%	92.40%	54%	65 bp	35.7
20180302.A-UP360miR_CLIPAgo2IP_3rd_R1	60.60%	30.2	69.70%	14.40%	91.60%	48%	67 bp	49.8
20180302.A-UP360miR_CLIPAgo2IP_4th_R1	60.60%	28.9	68.80%	14.10%	91.50%	48%	67 bp	47.7
20180302.A-UP360miR_CLIPinput_3rd_R1	85.40%	46.3	53.40%	9.30%	94.20%	51%	69 bp	54.2
20180302.A-UP360miR_CLIPinput_4th_R1	82.90%	42.5	54.90%	13.90%	92.80%	53%	66 bp	51.3
20180302.A-UP360miR_CLIPmiRCLIP_3rd_R1	40.20%	19.8	74.60%	17.40%	91.50%	48%	65 bp	49.3
20180302.A-UP360miR_CLIPmiRCLIP_4th_R1	36.90%	20.7	80.20%	14.90%	92.00%	47%	67 bp	56
BSSE_QGF_40194_C8112ANXX_6_UP358_input_ATCACGA_S1_L006_R1_001	79.00%	14.8	82.20%	8.90%	82.50%	56%	49 bp	18.7
BSSE_QGF_40195_C8112ANXX_6_UP360_input_CGATGTA_S2_L006_R1_001	73.90%	15.2	72.80%	14.90%	82.30%	52%	48 bp	20.5
BSSE_QGF_40196_C8112ANXX_6_mock_Ago_IP_TTAGGCA_S3_L006_R1_001	59.50%	12.7	87.40%	7.60%	30.30%	45%	50 bp	21.4
BSSE_QGF_40197_C8112ANXX_6_UP358_Ago_IP_TGACCAA_S4_L006_R1_001	69.00%	15	85.80%	13.30%	40.50%	46%	50 bp	21.8
BSSE_QGF_40198_C8112ANXX_6_UP360_Ago_IP_ACAGTGA_S5_L006_R1_001	61.60%	12.3	80.00%	12.90%	57.80%	49%	50 bp	19.9
BSSE_QGF_40199_C8112ANXX_6_UP358_miR_CLIP_GCCAATA_S6_L006_R1_001	50.80%	13.8	82.10%	12.30%	69.90%	49%	50 bp	27.2
BSSE_QGF_40200_C8112ANXX_6_UP360_miR_CLIP_CAGATCA_S7_L006_R1_001	46.90%	13.1	80.60%	15.70%	73.10%	49%	50 bp	27.8

Supplementary Table S5: QC parameters of the hp-132-2-sequenced libraries

Raw data uploaded to ArrayExpress under accession number E-MTAB-8517.

Formal name (in manuscript)	Experimental name
Hp-132-2	UP363

Sample Name	% mapped to transcript	Mapped reads	% overall Aligned	% Trimmed	% Duplicate reads	% GC	Length	Million sequences
20180302.A-UP363miR_CLIPAgo2IP_3rd_R1	47.50%	23	56.90%	14.50%	88.30%	51%	67 bp	48.3
20180302.A-UP363miR_CLIPinput_3rd_R1	76.80%	35	78.70%	15.50%	92.30%	56%	66 bp	45.6
20180302.A-UP363miR_CLIPmiRCLIP_3rd_R1	46.30%	18.9	73.80%	18.40%	90.30%	49%	66 bp	40.9
BSSE_QGF_60584_CAT8BANXX_5_UP363_input_1_ATCACGA_S1_L005_R1_001	53.50%	8.2	91.00%	4.00%	92.50%	55%	123 bp	15.3
BSSE_QGF_60585_CAT8BANXX_5_mock_Ago_IP_1_CGATGTA_S2_L005_R1_001	92.60%	19.4	96.10%	2.30%	71.70%	50%	125 bp	20.9
BSSE_QGF_60586_CAT8BANXX_5_UP_363_Ago_IP_1_TTAGGCA_S3_L005_R1_001	89.70%	18	95.20%	2.60%	74.50%	50%	125 bp	20.1
BSSE_QGF_60587_CAT8BANXX_5_UP363_miR_CLIP_1_TGACCAA_S4_L005_R1_001	43.50%	5.2	69.70%	10.90%	91.00%	51%	123 bp	11.9
BSSE_QGF_60588_CAT8BANXX_5_UP363_input_2_ACAGTGA_S5_L005_R1_001	60.70%	3.7	86.00%	34.00%	89.70%	53%	85 bp	6.1
BSSE_QGF_60589_CAT8BANXX_5_mock_Ago_IP_2_GCCAATA_S6_L005_R1_001	76.70%	7.5	85.80%	17.70%	87.20%	46%	106 bp	9.7
BSSE_QGF_60590_CAT8BANXX_5_UP_363_Ago_IP_2_CAGATCA_S7_L005_R1_001	48.90%	3.6	86.60%	29.50%	93.30%	49%	91 bp	7.3
BSSE_QGF_60591_CAT8BANXX_5_UP363_miR_CLIP_2_ACTTGAA_S8_L005_R1_001	59.20%	8	90.10%	12.10%	93.00%	49%	112 bp	13.5

Supplementary Table S6: Hp-124-1 captured transcripts

mRNAs captured by hp-124-1 and present in the top 1000 miR-CLIP/input enriched targets, bearing at least one target motif in their 3'UTR (miR-124 TargetScan 7m8 target motif, miR-124 G-bulge target motif, TargetScan iso-miR-124 7m8 target motif).

**hp-124-1 miR-124 7m8 seed targets (GTGCCTT)**	**hp-124-1 G-bulge targets (TGGCCTT)**	**hp-124-1 *iso*-miR-124 7m8 seed targets (TGCCTTA)**
AK2	ABCF1	ACAA2
AKAP13	AC011511.4	AKAP13
AMOTL1	AK2	AMOTL1
APEX2	AKAP13	ANP32E
ATP11A	AMOTL1	ANXA5
ATP2B4	ANK2	APBB2
AURKA	APBB2	APEX2
BCL2L11	ATG16L1	ARHGDIA
BRWD1	ATP11A	ATP11A
BTG2	ATP2B4	ATP2B4
C4orf46	BRWD1	AURKA
CAMTA1	CAMK2G	BAZ2B
CD164	CCDC14	BCL2L11
CDC14B	CD276	BRWD1
CERS2	CDC14B	C2orf15
CGN	CDC25A	C4orf46
CHIC1	CDC42SE2	CAMTA1
CREB3L2	CGN	CAMTA2
CTDSPL	CHD3	CAPN2
DAZAP2	CHD4	CD164
DCUN1D4	CPNE8	CD276
DDIAS	DDX52	CDC25A
DDX3X	DNMT1	CDC42SE2
DDX52	ELK4	CDK4
E2F3	ELL2	CENPQ
E2F5	EZR	CEP152
EIF3B	FYCO1	CERS2
EPC1	GATAD2A	CGN
GGA2	GOLGB1	CHEK1
HEATR1	HMBOX1	CHIC1
HIPK3	HMGA1	CPEB2
HIVEP1	HYOU1	CPS1
HIVEP2	IRF2BP2	CREB3L2
INTS6L	KCTD2	CSDE1
IQGAP1	KDM3B	CTDSPL
ITGB1	KIF1B	DAZAP2
JADE1	LAMTOR1	DCUN1D3
KANK1	MAGI3	DDX52
LAMC1	MARCH8	DIAPH1
LCOR	MEX3B	EFCAB14
LONRF1	MFSD9	ELK4

MAP3K21	MINK1	ELL2
MAPK7	MKL2	F11R
MARCH8	MRPS5	FAM222B
MGAT4A	MYH10	GALNT4
MGAT5	NR6A1	GATAD2A
MKL2	NSD1	GGA2
MPHOSPH9	NUFIP2	GIT2
MTR	NUMB	GOLGA3
MYADM	OCRL	HEATR1
MYH10	OXSR1	HIPK3
MYO10	PABPN1	HMGXB4
NAA15	PDPR	INTS6L
NEK6	PEAK1	IQGAP1
NFIA	PPARA	IRF2BP2
NFIB	PURA	JADE1
NFIC	RAB11FIP1	JAZF1
NKRF	RALGAPA1	KANK1
NLRX1	RAVER1	KIF23
NSD3	REPIN1	KLHL28
NUFIP2	RNF40	LAMC1
NUMA1	RORA	LARP1
PDE4D	RPL15	LCOR
PEAK1	RPLP0	LONRF1
PPARA	SAP130	LRRFIP1
PRR14L	SCRN1	LYRM2
PRR3	SERPINB6	MACF1
PTBP1	SF3B1	MAP3K21
RAB11FIP1	SLC37A4	MARCH8
RAPGEF1	SMARCA5	MED26
RAVER1	SOCS7	MGME1
RBMS1	SPART	MID1
RHOG	SRPK1	MPHOSPH6
ROCK1	TAB2	MTR
RRAGD	TBC1D14	MYADM
RREB1	TBC1D16	MYH10
SKP2	THOC2	MYLIP
SLC35A4	TNPO1	MYO10
SNAI2	TNRC6A	N4BP2
SNX9	TNRC6B	NAA15
SPECC1L	TP53INP1	NEK6
SUCO	TRIO	NFIB
SUPT16H	TROVE2	NFIC
TBC1D14	TRPC4AP	NHLRC3
TBC1D16	TTPAL	NLRX1
TBC1D20	UBE2V1	NOL4L
TEAD1	USP22	OTUD1
TNRC6B	VAMP3	OTUD4

TP53INP1	YOD1	OXSR1
TPD52L2	ZBTB18	PARP1
TRIP10	ZC3H4	PDE4D
TSC1	ZNF280B	PNN
TXNRD1	ZNF561	PPARA
UHMK1	ZNF678	PRR14L
UHRF1		PRUNE1
USP1		PTBP1
USP22		PTPN1
VAMP3		PURA
VAMP7		RAB11FIP1
WTAP		RAB6A
ZFAND3		RAI14
ZFP36L2		RAPGEF1
ZNF678		RAPGEF2
		RAVER1
		RBMS1
		RHOG
		ROCK1
		RPIA
		RRAGD
		RRBP1
		RREB1
		SLBP
		SLC50A1
		SMARCA5
		SNAI2
		SNX9
		SPECC1L
		SPRTN
		SUCLG2
		SURF4
		TAB2
		TACC1
		TARS
		TEAD1
		TGFBR3
		TP53INP1
		TPD52L2
		TRIM36
		TROVE2
		TXLNA
		VAMP3
		WASF1
		WDR45B
		YIPF6
		YOD1

		ZBTB7A
		ZCCHC14
		ZFP36L2
		ZNF140
		ZNF148
		ZNF35
		ZNF503
		ZNF559
		ZNF561
		ZNF678

Supplementary Table S7: Hp-124-3 captured transcripts

mRNAs captured by hp-124-3 and present in the top 1000 miR-CLIP/input enriched target, bearing at least one target motif in their 3'UTR (miR-124 TargetScan 7m8 target motif, miR-124 G-bulge target motif, TargetScan iso-miR-124 7m8 target motif).

**hp-124-3 miR-124 7m8 targets (GTGCCTT)**	**hp-124-3 G-bulge targets (TGGCCTT)**	**hp-124-3 *iso*-miR-124 7m8 targets (TGCCTTA)**
AFF1	AFF1	ABHD3
AKAP13	AK4	ACAA2
AMMECR1	AKAP13	AFF1
AMOTL1	AMOTL1	AK3
APEX2	ARL2	AK4
ATF7	ATF7	AKAP13
ATXN1L	ATP11A	AMMECR1
AURKA	BRWD1	AMOTL1
BRWD1	BTBD7	ANP32E
BTBD7	CACUL1	ANXA5
BTG2	CCDC14	APEX2
CACUL1	CCNG2	ARHGDIA
CADM1	CCNL2	ATF7
CAMTA1	CCNT1	ATP6V0E1
CCNG2	CDC25A	AURKA
CCNT1	CEP97	BAZ2B
CD164	CGN	BRWD1
CDC25B	CHD3	BTRC
CDON	CPNE8	C2orf15
CERS2	CTNND1	C9orf64
CGN	DENND1B	CAMTA1
CHIC1	DNMT1	CAMTA2
CKAP2L	ELK4	CCNT2
CLIP1	ENAH	CD164
CNEP1R1	EZR	CDC25A
CREB3L2	FAM53B	CDON
CTDSPL	FBXW11	CENPQ
CTNND1	FLNB	CERS2
CYBRD1	FYCO1	CGN
DDX3X	GATAD2A	CHIC1
DENND1B	GIGYF1	COMMD5
DYRK2	GOLGB1	CPS1
E2F3	HIPK2	CREB3L2
EIF4G1	HMGA1	CTDSPL
EPC1	HNRNPU	DCUN1D3
FAM53B	ILF3	DENND1B
FNBP1	INTS2	EFCAB14
FXR1	KDM2A	ELK4
G3BP1	KIDINS220	EPHA3
GGA2	LAMTOR1	ETFA
HIPK1	LMNB1	F11R

HIPK2	MFSD9	FAM222B
HNRNPU	MIGA1	FAM53B
HNRNPUL2	MINK1	FERMT2
ITGB1	MTF1	G3BP1
JADE1	MYH10	GALNT4
KANK1	NDE1	GATAD2A
L2HGDH	NR6A1	GGA2
LAMC1	NUDT4	GOLGA3
MAP2K4	NUFIP2	GPX8
MAP3K21	OCRL	GRB10
MAP4	OXSR1	GTF2H2C
MAPKAPK2	PABPC4L	HBP1
MGAT4A	PABPN1	HBS1L
MTR	PHACTR4	HIPK1
MYH10	PLEKHB2	HIPK2
MYO10	PPIL2	HMGXB4
NBPF9	PPP4R1	JADE1
NDE1	RAB11FIP1	KANK1
NFIA	RALGAPA1	KIF23
NLRX1	RAVER1	L2HGDH
NUFIP2	RGL1	LAMC1
P4HA1	RORA	LARP1
PABPC4L	RPL15	LRCH2
PDE4D	RPS10-NUDT3	LRRFIP1
PPP1R13L	SESN3	MAP2K4
PPP1R3B	SLBP	MAP3K21
PPP4R1	SOX6	MED26
PRKAG2	SPART	MID1
PRKD1	SRPK1	MIPOL1
PRR14L	TAB2	MPHOSPH6
PRTG	TAF4	MTF1
PTBP1	TBC1D14	MTR
PTPN12	TMBIM6	MYBL1
RAB11FIP1	TNPO2	MYH10
RAVER1	TNRC6A	MYLIP
RBM47	TNRC6B	MYO10
RC3H2	TP53INP1	N4BP2
RHOG	TRAF5	NHLRC3
ROCK1	TROVE2	NLRX1
RREB1	UBE2V1	NUDT4
SAMD4A	USP36	OTUD1
SESN3	WDR6	OTUD4
SETX	XYLB	OXSR1
SNAI2	YOD1	P4HA1
SNX9	ZBTB18	PABPC4L
SOCS5	ZC3H11A	PDE4D
SYPL1	ZC3H4	PNN

TBC1D14	ZNF280B	PPP1R13L
TBC1D20	ZNF473	PPP1R3B
TEAD1	ZNF561	PPP6R3
TNRC6B		PRKAG2
TP53INP1		PRKD1
TPD52L2		PRR14L
TRIP10		PRTG
TSC1		PRUNE1
TSKU		PTBP1
U2SURP		PTPN12
UHMK1		RAB11FIP1
USP1		RABGEF1
USP36		RAPGEF2
WNK1		RAVER1
ZFP36L2		RBM47
ZNF264		RHOG
		ROCK1
		RPIA
		RPS10-NUDT3
		RRBP1
		RREB1
		RUFY2
		SERTAD3
		SLBP
		SLC50A1
		SLC7A5
		SMC4
		SNAI2
		SNX9
		SP2
		SUCLG2
		SYPL1
		TAB2
		TBC1D9B
		TCF3
		TEAD1
		TGFBR3
		TP53INP1
		TPD52L2
		TROVE2
		TXLNA
		WASF1
		WDR45B
		YOD1
		ZBTB7A
		ZFP36L2
		ZNF140

		ZNF148
		ZNF264
		ZNF503
		ZNF559
		ZNF561
		ZNF562
		ZNF608

Supplementary Table S8: Properties of the top hp-124-3 captured, downregulated targets.

Relative expression of the top 16 most highly suppressed mRNAs from pre-miR-124-treated HEK293T cells, manually extracted from the Protein Atlas (www.proteinatlas.org).

Gene name	Highest expression score for brain tissues.	Link to the Protein Atlas
**APEX2**	N/A	https://www.proteinatlas.org/ENSG00000169188-APEX2/tissue
**ATP6V0E1**	N/A	https://www.proteinatlas.org/ENSG00000113732-ATP6V0E1/tissue
**SYPL1**	Low	https://www.proteinatlas.org/ENSG00000008282-SYPL1/tissue
**ITGB1**	Low	https://www.proteinatlas.org/ENSG00000150093-ITGB1/tissue
**LAMC1**	Low	https://www.proteinatlas.org/ENSG00000135862-LAMC1/tissue
**RAVER1**	Low	https://www.proteinatlas.org/ENSG00000161847-RAVER1/tissue
**CTDSPL**	Low	https://www.proteinatlas.org/ENSG00000144677-CTDSPL/tissue
**RHOG**	Low	https://www.proteinatlas.org/ENSG00000177105-RHOG/tissue
**POLR3G**	Low	https://www.proteinatlas.org/ENSG00000113356-POLR3G/tissue
**PPP1R13L**	Medium	https://www.proteinatlas.org/ENSG00000104881-PPP1R13L/tissue
**CD164**	Medium	https://www.proteinatlas.org/ENSG00000135535-CD164/tissue
**CREB3L2**	Medium	https://www.proteinatlas.org/ENSG00000182158-CREB3L2/tissue
**CERS2**	Medium	https://www.proteinatlas.org/ENSG00000143418-CERS2/tissue
**PTBP1**	Medium	https://www.proteinatlas.org/ENSG00000011304-PTBP1/tissue
**LMNB1**	High	https://www.proteinatlas.org/ENSG00000113368-LMNB1/tissue
**SNAI2**	High	https://www.proteinatlas.org/ENSG00000019549-SNAI2/tissue

Supplementary Table S9: Sequences of miR-124 duplexes

	Sequence 5'-3'
miR-124 guide strand	UAAGGCACGCGGUGAAUGCC
miR-124 passenger strand	UGUUCACCGCGUGCCUUGAU
Iso-miR-124 guide strand	UUAAGGCACGCGGUGAAUGCC
Iso-miR-124 passenger strand	UGUUCACCGCGUGCCUUGAUU

Supplementary Table S10: Properties of the top hp-124-3 captured, downregulated targets.

Numbers of 7merA1, 7m8 and 8mer broadly conserved, predicted sites extracted from the longest representative most prevalent transcripts from TargetScan (http://www.targetscan.org/vert_72/; Release 7.2, March 2018) for the top 16 most highly suppressed mRNAs from pre-miR-124-treated HEK293T cells.

**Target**	**miR-124**<br>7merA1<br>TGCCTTA	 7m8<br>GTGCCTT	 8mer<br>GTGCCTTA		***Iso*-miR-124**<br>7merA1<br>GCCTTAA	 7m8<br>TGCCTTA	 8mer<br>TGCCTTAA
**ITGB1**		2					
**SNAI2**		2	1				
**ATP6V0E1**	2					1	1
**PPP1R13L**	1	1			1	1	
**CD164**			2			1	1
**SYPL1**	1	1					
**APEX2**			1		1		1
**LAMC1**	1	2					1
**CREB3L2**			2				2
**RAVER1**	1	1			1		1
**CERS2**			1			1	
**PTBP1**	2		1			2	1
**CTDSPL**	2	1				1	1
**LMNB1**					1		
**RHOG**		3	1				1
**POLR3G**	1		1			1	1

Supplementary Table S11: Selective inhibition of proteins containing G-bulged sites by miR-124 (separate Excel file).

The first worksheet in the Excel file contains calculated expression change per protein together with statistics. The second worksheet contains more detailed peptide data used for targeted detection of the nine proteins.

Supplementary Methods

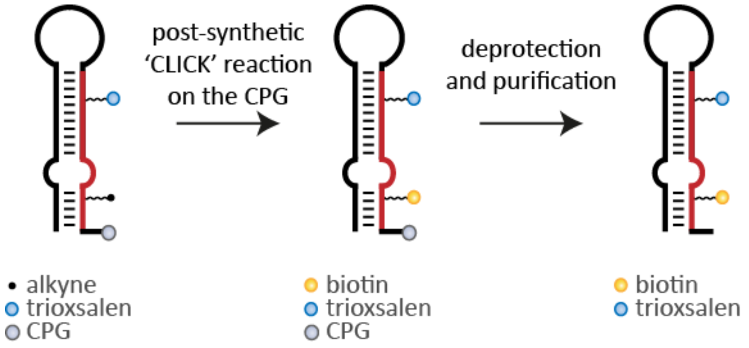

Scheme S1. Preparation of trioxsalen (psoralen)/biotin bis-labeled pre-miR-CLIP probes.

Chemical synthesis

4'-azidomethyltrioxsalen, 2'-O-propargyl-N^4-benzoyladenosine phosphoramidite, 2'-O-trioxsalen-N^4-benzoyladenosine phosphoramidite, 2'-O-propargyl-N^4-acetylcytidine phosphoramidite and azido-TEG-biotin were synthesized according to the published procedures.(2,3)

Oligonucleotide synthesis, deprotection and purification

Chemicals were purchased from Biosolve, Sigma-Aldrich, VWR, Acros Organics and TCI. Phosphoramidites were purchased from Thermo Fisher. The activator 5-benzylthio-1H-tetrazole was purchased from Carbosynth. All oligonucleotides used in this work were synthesized on a 50 nmol or 1 µmol scale with the MM12 synthesizer (Bio Automation Inc.) using 500 Å or 2000 Å UnyLinker Controlled Pore Glass (CPG; ChemGenes).

The coupling time of phosphoramidites was 2 x 180 s (50 nmol scale) or 1 x 240 s (1 µmol scale) for standard phosphoramidites and 2 x 240 s (50 nmol scale) or 1 x 360 s (1 µmol scale) for modified phosphoramidites.

After the solid phase synthesis, the CPG was treated with gaseous methylamine for 1.5 h at 70 °C (unmodified oligoribonucleotides) or with a mixture of 200 µL of ammonia solution (25% in H_2O) and 200 µL of methylamine solution (40% in H_2O) for 6 h at 35 °C (modified oligoribonucleotides). After basic deprotection and cleavage from the solid support, the CPG was washed with 3 x 200 µL of H_2O/EtOH (1:1) mixture. To the collected filtrate 20 µL of 1 M Tris-base was added and it was evaporated to dryness in a SpeedVac. Desilylation was carried out by treatment with 130 µL of a freshly prepared mixture of NMP (60 µL), TEA (30 µL) and TEA x 3HF (40 µL) at 70 °C for 2 h. The reaction was quenched with trimethylethoxysilane (200 µL, 3 min, room temperature). Diethyl ether (200 µL) was then added and the mixture was vortexed (5 min, room temperature) and centrifuged.

In case of the 1 µmol synthesis, the CPG was divided into smaller portions (around 20 mg of the CPG each) and treated with a doubled number of reagents.

The precipitate was dissolved in 200 µL of H_2O and purified on an Agilent 1200 series preparative RP-HPLC with an XBridge OST C-18 column (10 x 50 mm, 2.5 µm; Waters) at 65 °C with a flow rate 5 mL/min. Solvent A: 0.1 M aqueous triethylamine/acetic acid, pH 8.0; Solvent B: 100% MeOH; gradient 20-60% B in 5 min.

Fractions containing the DMTr-on product were collected, dried in a SpeedVac and treated with 40% aqueous acetic acid for 15 min at room temperature. Samples were concentrated in a SpeedVac, dissolved in 200 µL of H_2O and purified in DMT-off mode on an Agilent 1200 series preparative RP-HPLC with an XBridge OST C-18 column (10 x 50 mm, 2.5 µm; Waters) at 65 °C with a flow rate 5 mL/min. Solvent A: 0.1 M aqueous triethylamine/acetic acid, pH 8.0; Solvent B: 100% MeOH; gradient 5-30% B in 5 min.

Fractions containing the product were collected, dried in a SpeedVac, re-dissolved in 200 µL of H_2O and analysed by LC-MS (Agilent 1200/6130 system) on an Acquity OST C-18 column (2.1 x 50 mm; Waters). The column oven was set to 65 °C, flow-rate: 0.3 mL/min. Solvent C: H_2O containing 0.4 M HFIP, 15 mM triethylamine; solvent B: MeOH; gradient 5-40% B in MeOH (pre-miR-124), 5-50% B in 14 min (hp-124-1, hp-124-3, ctstr-132-GNL3L, gs-132-2), 5-35% B in 14 min (hp-132-2) or 1-35% B in 10 min (hp-132-1).

UV-absorption of the final products was measured in duplicate (modified oligoribonucleotides) or triplicate (unmodified oligoribonucleotides) on a NanoDrop 2000 spectrophotometer (Fisher Scientific). Concentrations were calculated based on the nearest neighbour model with in-house programmed software.

MiR-CLIP protocol

Cell transfection and lysis: Method was adapted from that described in reference (2). 5% Of the fully confluent HEK293T cells from a T150 flask were seeded into 15 cm dishes. 4 h after seeding, miR-CLIP probes were transfected with Lipofectamine RNAiMAX (13778150, Thermo Fisher Scientific) according to the manufacturer's protocol. MiR-CLIP probes were transfected at final concentrations of 10 nM for hp-124-1 and hp-124-3, or 2.5 nM for hp-132-2. Treatment containing transfection reagent without RNA was used as a negative control.

Cells were put on ice, washed with 1x PBS and irradiated twice at 254 nm with 100 mJ (Ultraviolett Cross Linker 254 nm, UVP). Then, cells were irradiated twice at 365 nm with 150 mJ (BLX-254 Bio-Link cross-linker (VilberLourmat) equipped with 365 nm lamps). After the irradiations, cells were scrapped with PBS, pelleted at 200g, 4°C for 5 min and lysed with 1 mL NP40 lysis buffer (50 mM HEPES pH 7.5, 150 mM KCl, 0.5 mM DTT, 2 mM EDTA, 50 U/mL RNAsin, 1x complete protease inhibitor (Roche)) for 15 min on ice. The lysate was cleared for 15 min at 14'000 x g at 4°C. The input samples (25 µL each tube) were removed at this stage.

AGO2 Immunoprecipitation: Per each 15 cm dish, 100 µL of Dynabead™ Protein G were prepared according to manufacturer's instructions. The AGO2 antibody (clone 11A9, Ascenion, 50 µg per 100 µL of Prot G Dynabeads) was immobilized in a total volume of 500 µL citrate-phosphate buffer (25 mM citric acid, 66 mM Na_2HPO_4, pH 5.0) by gentle rolling for 1 h at 4°C. The beads were washed with 1 mL of NP40 lysis buffer (50 mM HEPES pH 7.5, 150 mM KCl, 0.5 mM DTT, 2 mM EDTA, 50 U/mL RNAsin, 1x complete protease inhibitor (Roche)) and blocked for 1 h at 4°C with 1 mL of NP40 lysis buffer containing BSA (10 µg/mL). Beads were washed with 1 mL of NP40 lysis buffer and resuspended in 50 µL.

Lysate was incubated with the AGO2 antibody-coupled Protein G beads for 1 h at 4° C with gentle rolling. Beads were washed 5x with 1 mL IP wash buffer (50mM HEPES, pH 7.5, 300 mM KCl, 0.05 % NP40, 0.5 mM DTT, 1x cOmplete).

After the last washing step, 200 µL of digestion buffer (100 mM Tris-HCl pH 7.5, 150 mM NaCl, 12.5 mM EDTA) containing 12 U proteinase K (03115828001, Roche) was added to each sample and the proteins were digested for 15 min at 65°C. 1.33 µL Glycoblue (AM9515, Thermo Fisher Scientific) was added to each tube. RNA from the proteinase K digestion was isolated using acid-phenol:chloroform extraction (AM9720, Thermo Fisher Scientifics) followed by overnight ethanol precipitation at -20°C. After precipitation, the RNA pellet was washed with 70 % EtOH, centrifuged at 12.000 x g for 5 min at 4°C and dissolved in 20µL of water. At this point, 5% of the sample was removed as AGO2 IP fraction after precipitation.

Streptavidin-biotin affinity purification: Per each 15 cm dish, 20 µL of magnetic Dynabeads™ MyOne™ Streptavidin C1 (65001, Life Technologies) were washed according to the manufacturer's protocol and blocked with 1 mL of binding and washing buffer (B&W-buffer, 0.5 mM EDTA, 1.0 mM NaCl, 50 U/mL RNAsin, 0.001% NP40, pH 7.5) containing salmon sperm DNA (100 µg/mL), BSA (100 µg/mL) and heparin (0.1 µg/mL) for 1 h at 4°C. Beads were washed 3x 1 mL of B&W buffer and pre-equilibrated in 500 µLNP40 lysis buffer.

For streptavidin affinity purification, the RNA was redissolved in 20 µL of B&W buffer and incubated with streptavidin beads for 30 min at 4°C with gentle rolling. Beads were washed 5x with 1 mL B&W

buffer, treated with 1 µLRQ1 DNAse/dish in the appropriate amount of buffer (M6101, Promega) for 15 min at 37°C and washed with 1 mL B&W buffer.

The beads were treated two times with 50 µL of a solution of 95% formamide/5% 10 mM aq. EDTA pH 8.2 for 5 min at 65°C. The solutions were combined, and diluted to 100 µL with water. 1.33 µL Glycoblue was added to each tube. The RNA was purified by acid:chloroform/phenol (Life Technologies) extractions and the RNA was precipitated as described above. 1 µL of the sample was removed for the in-line control, RT-qPCR as described above.

The RNAs were Ribo-depleted using the Ribo-zero kit from Epicentre (now Illumina, discontinued) according to the manufacturer's instructions. RNA was fragmented using alkaline hydrolysis buffer (50 mM $NaCO_3$, 1 mM EDTA, pH 9.2) by heating at 95°C for 4 min and then put on ice. Preparation of the first libraries for hp-124-1, hp-124-3 and hp-132-2 was carried out according to ref (2). The remaining libraries were dephosphorylated and phosphorylated before they were subjected to the CleanTag® small RNA Library Prep Kit (L-3206, TriLink).

Small RNA sequencing

Pre-miR-124 was transfected using Lipofectamine 2000 as described above. Cells were collected 48 h after transfection, using the miRVana™ miRNA Isolation Kit (AM1560, Thermo Fisher Scientific) applying the "Enrichment Procedure for Small RNAs according to the manufacturer's protocol. The small RNA fraction was concentrated by precipitation and sequencing library prepared using the CleanTag® small RNA Library Prep Kit (L-3206, TriLink). PCR products were cleaned up using Agencourt AMPure XP kit according to the manufacturer's protocol (A63880, Beckman-Coulter).

Sequencing analysis

3' Adapters were removed from all small RNA sequencing libraries with cutadapt v1.16(4), passing the 3' adapter sequence "TGGAATTCTCGGGTGCCAAGG" to the –adapter parameter, as well as the following non-default arguments: --max-n=1 and --minimum-length=14. Identical sequences were collapsed. The libraries were then aligned to the human genome (Ensembl GRCh38 primary assembly, soft-masked) and transcriptome (Ensembl release 89)(5) with segemehl v0.2.0(6) and default parameters. Reads not exceeding 30 nucleotides in length after adapter trimming were also aligned to transcriptome and genome with oligomap v1.0(7) to make use of its non-heuristic, high-fidelity alignment approach. Transcriptome alignments were then processed and converted to genome coordinates with custom scripts, as previously described(8) (custom scripts are available at https://github.com/zavolanlab/IsoformQuantificationBenchmarking). Briefly, sense alignments to transcriptomes were migrated from transcriptome to genome coordinate space with the help of gene annotations (Ensembl release 89) and then merged with the genomic alignments. The resulting set of four separate sets of alignments (originating from transcriptome and genome alignments computed with segemehl and oligomap) were merged and duplicate and inferior alignments were discarded, retaining only the best possible alignments (i.e., those with the smallest edit distances) for each read. Finally, the merged and filtered alignments were sorted and compressed with SAMtools v1.8(9) to generate BAM files. MicroRNA counts were obtained by intersecting the alignments with annotations from miRBase(10), release 22, with the help of the Bioconductor suite(11).

Shotgun LC-MS experiments

Quantitative proteomics of miR-124-, isomiR-124- and control-transfected cells were performed as described in ref(12). Briefly cells were lysed in a lysis buffer including 1% sodium deoxycholate, 10mM TCEP, 100mM Tris, pH=8.5 and sonicated in a Bioruptor (Dianode) and subsequently alkylated with chloroacetamide. The extracted proteins were then quantified with a reducing agent compatible BCA assay (Pierce #23252).100 µg of protein from each sample was then used for further protein digestion with Trypsin and subsequent inhibition with TFA. The digested peotides were then extracted with a C18 column based by solid phase extraction. Peptides were quantified and 20 µg of each sample was then used for multiplexing TMT reagents from ThermoFisher Scientific. To control for ratio distortion during quantification, a peptide calibration mixture consisting of six digested standard proteins mixed in different amounts were added to each sample before TMT labeling. After pooling the TMT labeled peptide samples, peptides were again desalted on C18 reversed-phase spin columns according to the

manufacturer's instructions (Macrospin, Harvard Apparatus) and dried under vacuum. TMT-labeled peptides were fractionated by high-pH reversed phase separation using a XBridge Peptide BEH C18 column (3,5 µm, 130 Å, 1 mm x 150 mm, Waters) on an Agilent 1260 Infinity HPLC system. Peptides were loaded on column in buffer A (ammonium formate (20 mM, pH 10) in water) and eluted using a two-step linear gradient starting from 2% to 10% in 5 minutes and then to 50% (v/v) buffer B (90% acetonitrile / 10% ammonium formate (20 mM, pH 10) over 55 minutes at a flow rate of 42 µl/min. Elution of peptides was monitored with a UV detector (215 nm, 254 nm). A total of 36 fractions were collected, pooled into 12 fractions using a post-concatenation strategy as previously described(13), dried under vacuum and subjected to LC-MS/MS analysis.

Targeted PRM-LC-MS analysis

In a first step, parallel reaction-monitoring (PRM) assays(14) were generated from a mixture containing 100 fmol of each heavy reference peptide and shotgun data-dependent acquisition (DDA) LC-MS/MS analysis on a Thermo Orbitrap Fusion Lumos platform (Thermo Fisher Scientific). The setup of the µRPLC-MS system was as described previously(15). Chromatographic separation of peptides was carried out using an EASY nano-LC 1200 system (Thermo Fisher Scientific), equipped with a heated RP-HPLC column (75 µm x 30 cm) packed in-house with 1.9 µm C18 resin (Reprosil-AQ Pur, Dr. Maisch). Peptides were analyzed per LC-MS/MS run using a linear gradient ranging from 95% solvent A (0.15% formic acid, 2% acetonitrile) and 5% solvent B (98% acetonitrile, 2% water, 0.15% formic acid) to 45% solvent B over 60 minutes at a flow rate of 200 nl/min. Mass spectrometry analysis was performed on Thermo Orbitrap Fusion Lumos mass spectrometer equipped with a nanoelectrospray ion source (both Thermo Fisher Scientific). Each MS1 scan was followed by high-collision-dissociation (HCD) of the 10 most abundant precursor ions with dynamic exclusion for 20 seconds. Total cycle time was approximately 1 s. For MS1, 1e6 ions were accumulated in the Orbitrap cell over a maximum time of 100 ms and scanned at a resolution of 120,000 FWHM (at 200 m/z). MS2 scans were acquired at a target setting of 1e5 ions, accumulation time of 50 ms and a resolution of 30,000 FWHM (at 200 m/z). Singly charged ions and ions with unassigned charge state were excluded from triggering MS2 events. The normalized collision energy was set to 30%, the mass isolation window was set to 1.4 m/z and one microscan was acquired for each spectrum.

The acquired raw-files were database searched against a human database (Uniprot, download date: 2017/10/23 total of 20,179 entries) by the MaxQuant software (Version 1.623). The search criteria were set as following: full tryptic specificity was required (cleavage after lysine or arginine residues); 3 missed cleavages were allowed; carbamidomethylation (C) was set as fixed modification; Arg10 (R), Lys8 (K) and oxidation (M) as variable modification. The mass tolerance was set to 10 ppm for precursor ions and 0.02 Da for fragment ions. The best 6 transitions for each peptide were selected automatically using an in-house software tool. A mass isolation lists containing all selected peptide ion masses were split into 8 mass lists by charge, C-terminal amino acid and transition and precursor ions. The mass lists were then imported into the Lumos operating software for SureQuant analysis using the following settings: The resolution of the orbitrap was set to 30k (120k) FWHM (at 200 m/z) for heavy (light) peptide ions and the fill time was set to 54 (246) ms, respectively, to reach a target value of 1e6 ions. Ion isolation window was set to 0.4 Th and the scan range was set to 150-1500 Th. The mass window for triggering heavy PRM scans was set to 10 ppm and the depended PRM triggering threshold for the light channel was set to a minimum of 2 detected transitions. A MS1 scan using the same conditions are for DDA was included in each MS cycle. Each condition was analyzed in biological triplicates.

PRM data analysis

All raw-files were imported into Spectrodive software (version 10.0.200312) and processed using the standard SureQuant settings. Only peptides with an elution group q value < 0.01 were considered for quantitative analysis. The transition group raw intensities (TG.Raw.Intensity) of light channel were exported, log2 transformed and used for quantification. To control for variation in sample amounts, the total ion chromatogram (only comprising peptide ions with two or more charges) of each sample was determined by Progenesis QI (version 2.0, Waters) and used for normalization.

References

1. Patro, R., Duggal, G., Love, M.I., Irizarry, R.A. and Kingsford, C. (2017) Salmon provides fast and bias-aware quantification of transcript expression. *Nat Methods*, **14**, 417-419.
2. Imig, J., Brunschweiger, A., Brümmer, A., Guennewig, B., Mittal, N., Kishore, S., Tsikrika, P., Gerber, A.P., Zavolan, M. and Hall, J. (2014) miR-CLIP capture of a miRNA targetome uncovers a lincRNA H19–miR-106a interaction. *Nature Chemical Biology*, **11**, 107.
3. Pradere, U., Brunschweiger, A., Gebert, L.F., Lucic, M., Roos, M. and Hall, J. (2013) Chemical synthesis of mono- and bis-labeled pre-microRNAs. *Angew Chem Int Ed Engl*, **52**, 12028-12032.
4. Martin, M. (2011) Cutadapt removes adapter sequences from high-throughput sequencing reads. *EMBnet.journal; Vol 17, No 1: Next Generation Sequencing Data AnalysisDO - 10.14806/ej.17.1.200*.
5. Cunningham, F., Achuthan, P., Akanni, W., Allen, J., Amode, M.R., Armean, I.M., Bennett, R., Bhai, J., Billis, K., Boddu, S. *et al.* (2019) Ensembl 2019. *Nucleic Acids Res*, **47**, D745-D751.
6. Otto, C., Stadler, P.F. and Hoffmann, S. (2014) Lacking alignments? The next-generation sequencing mapper segemehl revisited. *Bioinformatics*, **30**, 1837-1843.
7. Berninger, P., Gaidatzis, D., van Nimwegen, E. and Zavolan, M. (2008) Computational analysis of small RNA cloning data. *Methods*, **44**, 13-21.
8. Kanitz, A., Gypas, F., Gruber, A.J., Gruber, A.R., Martin, G. and Zavolan, M. (2015) Comparative assessment of methods for the computational inference of transcript isoform abundance from RNA-seq data. *Genome Biol*, **16**, 150.
9. Li, H., Handsaker, B., Wysoker, A., Fennell, T., Ruan, J., Homer, N., Marth, G., Abecasis, G., Durbin, R. and Genome Project Data Processing, S. (2009) The Sequence Alignment/Map format and SAMtools. *Bioinformatics*, **25**, 2078-2079.
10. Kozomara, A., Birgaoanu, M. and Griffiths-Jones, S. (2019) miRBase: from microRNA sequences to function. *Nucleic Acids Res*, **47**, D155-D162.
11. Huber, W., Carey, V.J., Gentleman, R., Anders, S., Carlson, M., Carvalho, B.S., Bravo, H.C., Davis, S., Gatto, L., Girke, T. *et al.* (2015) Orchestrating high-throughput genomic analysis with Bioconductor. *Nat Methods*, **12**, 115-121.
12. Ahrné, E., Glatter, T., Viganò, C., Schubert, C.v., Nigg, E.A. and Schmidt, A. (2016) Evaluation and Improvement of Quantification Accuracy in Isobaric Mass Tag-Based Protein Quantification Experiments. *Journal of Proteome Research*, **15**, 2537-2547.
13. Wang, Y., Yang, F., Gritsenko, M.A., Wang, Y., Clauss, T., Liu, T., Shen, Y., Monroe, M.E., Lopez-Ferrer, D., Reno, T. *et al.* (2011) Reversed-phase chromatography with multiple fraction concatenation strategy for proteome profiling of human MCF10A cells. *PROTEOMICS*, **11**, 2019-2026.
14. Peterson, A.C., Russell, J.D., Bailey, D.J., Westphall, M.S. and Coon, J.J. (2012) Parallel reaction monitoring for high resolution and high mass accuracy quantitative, targeted proteomics. *Mol Cell Proteomics*, **11**, 1475-1488.
15. Ahrné, E., Glatter, T., Viganò, C., Schubert, C., Nigg, E.A. and Schmidt, A. (2016) Evaluation and Improvement of Quantification Accuracy in Isobaric Mass Tag-Based Protein Quantification Experiments. *J Proteome Res*, **15**, 2537-2547.
